# Awake Prone Positioning in Patients With COVID-19 Respiratory Failure: A Randomized Clinical Trial

**DOI:** 10.1001/jamanetworkopen.2025.48201

**Published:** 2025-12-10

**Authors:** Anatole Harrois, Romain Jouffroy, Soufia Ayed, Cédric Bruel, Laurent Savale, Mathilde Devaux, Mylène Maillet, Charles Cerf, Clément Lejealle, Carolina Gomez Moreno, Albrice Levrat, Romain Gueneau, Stéphane Jouveshomme, Pierre Bonnin, Stéphane De Rudnicki, Charles Damoisel, Thomas Gille, Hugues Cordel, Simon Meslin, Victor Bocquillon, Nicolas Noël, Antoine Vieillard-Baron, Jean-Louis Teboul, Marc Tran, Marie Werner, Jérémie Pichon, Ali Janbain, Eric Vicaut, Jacques Duranteau

**Affiliations:** 1 Service d’Anesthésie Réanimation Chirurgicale, DMU 12 Anesthésie Réanimation Chirurgicale Médecine Péri-Opératoire et Douleur, Hôpital Bicêtre, Assistance Publique–Hôpitaux de Paris (AP-HP), Université Paris-Saclay, Équipe DYNAMIC, Institut National de la Santé et de la Recherche Médicale (INSERM) UMR_S999, Le Kremlin-Bicêtre, France; 2 Medical Intensive Care Unit, Ambroise Paré Hospital, AP-HP, Boulogne-Billancourt, France; 3 INSERM UMR 1018, Clinical Epidemiology Team, Centre de Recherche en Epidémiologie et Santé des Populations (CESP), Université de Paris Saclay, Villejuif, France; 4 Service de Médecine Intensive-Réanimation, AP-HP, Hôpital de Bicêtre, DMU 4 CORREVE, INSERM UMR S_999, FHU SEPSIS, CARMAS, Université Paris-Saclay, Le Kremlin-Bicêtre, France; 5 Medical and Surgical Intensive Care Unit, Groupe Hospitalier Paris Saint Joseph, Paris, France; 6 AP-HP, Centre de Référence de l’Hypertension Pulmonaire, Service de Pneumologie et Soins Intensifs Respiratoires, Hôpital Bicêtre, Le Kremlin-Bicêtre, France; 7 INSERM UMR_S 999, Hypertension Pulmonaire: Physiopathologie and Innovation Thérapeutique (HPPIT), AP-HP, Hôpital Bicêtre, Hôpital Marie Lannelongue (Groupe Hospitalier Paris Saint Joseph), ERN-LUNG, Le Kremlin-Bicêtre, France; 8 Internal Medicine, Saint-Germain-en-Laye Intercommunal Hospital Center, Poissy, France; 9 Service de Maladies Infectieuses–Médecine Interne, Centre Hospitalier Annecy Genevois, Épagny Metz-Tessy, France; 10 Polyvalent Intensive Care Unit, Hôpital Foch, Suresnes, France; 11 Réanimation Médico-Chirurgicale, Hôpital Avicenne, AP-HP, Université Sorbonne Paris Nord, Bobigny, France; 12 INSERM UMR_S1155, Common and Rare Kidney Diseases (CORAKID), Hôpital Tenon, Sorbonne Université, Paris, France; 13 Department of Emergency Medicine, Instituto Nacional de Ciencias Medicas y Nutricion Salvador Zubiran, Mexico City, Mexico; 14 Anesthesiology and Critical Care Department, Annecy Hospital, Annecy, France; 15 Department of Infectious Diseases, Hôpital de Bicêtre, AP-HP, Le Kremlin-Bicêtre, France; 16 Service de Pneumologie, Groupe Hospitalier Paris Saint-Joseph, Paris, France; 17 Department of Infectiology, Annecy Genevois Hospital, Metz-Tessy, France; 18 Federation of Anesthesiology, Intensive Care Unit, Burns and Operating Theater, Percy Military Training Hospital, Clamart, France; 19 Service de Réanimation Polyvalente, Hôpital Antoine Béclère, AP-HP, Clamart, France; 20 INSERM U1272 Hypoxia and the Lung, Université Sorbonne Paris Nord, Bobigny, France; 21 Hôpitaux Universitaires de Paris Seine-Saint-Denis, sites Avicenne et Jean Verdier, Service Pneumologie et Physiologie/Explorations Fonctionnelles, AP-HP, Bobigny, France; 22 Infectious Disease Department, Avicenne Hospital, Hôpitaux Universitaires Paris Seine-Saint-Denis, AP-HP, Bobigny, France; 23 Université Sorbonne Paris Nord, Laboratoire Éducations et Pratiques de Santé, LEPS EA 3412, Paris, France; 24 Anaesthesiology and Critical Care Medicine Department, Hôpital Européen Georges Pompidou, AP-HP, Paris, France; 25 Department of Pneumology, Annecy Genevois Hospital, Metz-Tessy, France; 26 AP-HP, Université Paris-Saclay, Hôpital Bicêtre, Service de Médecine Interne et Immunologie Clinique, Le Kremlin-Bicêtre, France; 27 Department of Biostatistics, AP-HP, Université Paris-Diderot, Sorbonne-Paris Cité, Fernand Widal Hospital, Paris, France

## Abstract

**Importance:**

Awake prone positioning (APP) has shown inconstant associations with improved clinical outcomes in nonintubated patients with COVID-19 developing severe pneumonia.

**Objective:**

To evaluate the effects of APP on the need for intubation or incidence of death among patients with COVID-19–related hypoxemic respiratory failure.

**Design, Setting, and Participants:**

This randomized clinical trial was conducted at 20 hospitals in France and 1 hospital in Mexico between July 2020 and August 2021. The study included patients from wards and intensive care units. Adult patients (18 years or older) who were not intubated and required at least 3 L/min of oxygen flow due to COVID-19 infection were included and randomly assigned in a 1:1 ratio to either APP or standard care. Intention-to-treat statistical analysis was performed from September to December 2024.

**Intervention:**

Patients randomly assigned to the APP group were offered the intervention lasting at least 6 hours a day. Patients randomly assigned to standard care had no positioning constraint, including no contraindication to spontaneous APP.

**Main Outcomes and Measures:**

The primary outcome was a composite criterion of intubation and/or death in the first 28 days of randomization. Prespecified secondary outcomes at 28 days of enrollment were days alive outside the intensive care unit (ICU), days alive outside the hospital, proportion of patients admitted to ICU (for patients not in ICU at baseline), and days alive and free from mechanical ventilation. A bayesian approach was used to provide insights into the complete distribution of the effect estimates.

**Results:**

A total of 445 patients were included in the final analysis (mean [SD] age, 60 [11] years; 329 males [74%]; median [IQR] SpO_2_ to FIO_2_ [peripheral oxygen saturation to fraction of inspired oxygen] ratio, 150 [114-194] and 155 [109-221] in the standard care and APP groups, respectively). With a noninformative prior distribution, the posterior probability that APP decreased intubation and/or death compared with standard care was 93.8% (mean odds ratio [OR], 0.74; 95% credible interval [CrI], 0.48-1.09). For secondary outcomes, between the APP and standard care groups, the mean difference in the number of days alive and free from mechanical ventilation was 0.33 (95% CrI, −1.37 to 2.03) days; in the number of days alive outside the ICU was 1.28 (95% CrI, −0.78 to 3.34) days; and in the number of days alive outside the hospital was 1.55 (95% CrI, −0.22 to 3.32) days.

**Conclusions and Relevance:**

In this randomized clinical trial of nonintubated patients with COVID-19 and hypoxemic respiratory failure, daily APP of 6 hours showed a high probability of reduced endotracheal intubation and/or death over a wide range of prior distributions. These results support APP’s use in patients with hypoxemic pneumonia due to COVID-19 infection.

**Trial Registration:**

ClinicalTrials.gov Identifier: NCT04366856

## Key Points

**Question:**

What are the effects of awake prone positioning on the incidence of intubation and/or death in nonintubated patients with COVID-19 and hypoxemic respiratory failure?

**Findings:**

In this randomized clinical trial of 445 patients, the posterior probability that APP decreased intubation and/or death over the first 28 days of admission compared with standard care was high at 93.8%.

**Meaning:**

Findings of this trial support the use of awake prone positioning in patients with hypoxemic pneumonia due to COVID-19 infection.

## Teaser

This randomized clinical trial examines the incidence of intubation and/or death and other outcomes among patients with COVID-19–related hypoxemic respiratory failure receiving daily awake prone positioning or standard care.

## Introduction

Hypoxemic respiratory failure develops in the most severe form of COVID-19 infection and may ultimately turn into acute respiratory distress syndrome (ARDS), with the need for mechanical ventilation. In patients with ARDS under mechanical ventilation, prone positioning for at least 16 hours daily showed an improvement in survival.^1^ In patients not intubated developing hypoxemic respiratory failure, awake prone positioning (APP) was reported to improve oxygenation, respiratory rate, and cardiac performances and promote tidal volume distribution toward dorsal lung regions.^2,3^

Early in the course of the COVID-19 pandemic, APP was applied to improve oxygenation while critical care resources were limited due to surges in severe patients’ flow.^4^ Two large randomized studies assessed the effects of APP in patients with COVID-19 infection experiencing severe hypoxemic respiratory failure. The first study, a meta-trial of 6 randomized clinical trials (RCTs), reported a decrease in intubation and/or death mainly based on a reduction in intubation rate, while the second study reported no decrease in intubation rate.^5,6^ Meta-analyses aggregating RCTs reported a substantial decrease in intubation rate and an increase in survival.^7,8,9,10^ The European Society of Intensive Care Medicine guidelines on ARDS suggest applying APP in nonintubated patients to reduce intubation but do not make a recommendation for or against using APP to reduce mortality.^11^

The aim of the PROVID study was to assess the effects of APP on the need for intubation or incidence of death among patients with COVID-19–related hypoxemic respiratory failure. We used a bayesian approach because it provides insights into the complete distribution of the effect estimates. For instance, with those distributions, the posterior probability of a more than trivial benefit (odds ratio [OR] <0.95) and a more than trivial harm (OR >1.05) for a binary outcome can be calculated. For quantitative outcomes, the posterior probability of a benefit or harm corresponding to 1 or 2 days’ difference between groups can also be calculated. This approach contrasts with traditional point estimates associated with a single *P* value calculation and, we believe, provides a more complete and clinically meaningful interpretation of the results of a trial.

## Methods

### Trial Design and Oversight

We conducted a multicenter RCT at 20 centers in France and 1 center in Mexico. The trial protocol (Supplement 1) was approved by the South East II Ethics Committee at the Groupement Hospitalier Est (Hospices Civils de Lyon) for the French centers and by the Ethics Committee at the Instituto Nacional de Ciencias Medicas y Nutricion Salvador Zubiran for the Mexican center. We obtained written informed consent from all participating patients before randomization. The reporting of this trial adhered to the Consolidated Standards of Reporting Trials (CONSORT) reporting guideline.

### Patients

Inclusion criteria were age 18 years or older, COVID-19 infection (positive polymerase chain reaction test result and/or typical lung infiltrate on computed tomography scan), and requirement of at least 3 L/min of oxygen flow to get a peripheral oxygen saturation (SpO_2_) superior or equal to 95%. Exclusion criteria were age older than 80 years, pregnancy, inability to be in the prone position, and decision to withdraw life-sustaining treatments. Initially, patients with a do-not-intubate order were not included in the study. However, given that proning could be beneficial in that setting, we (South East II Ethics Committee) made an amendment. Ultimately, patients with a do-not-intubate order could be included when all therapies but intubation were still considered.

### Randomization and Intervention

**Figure 1.**
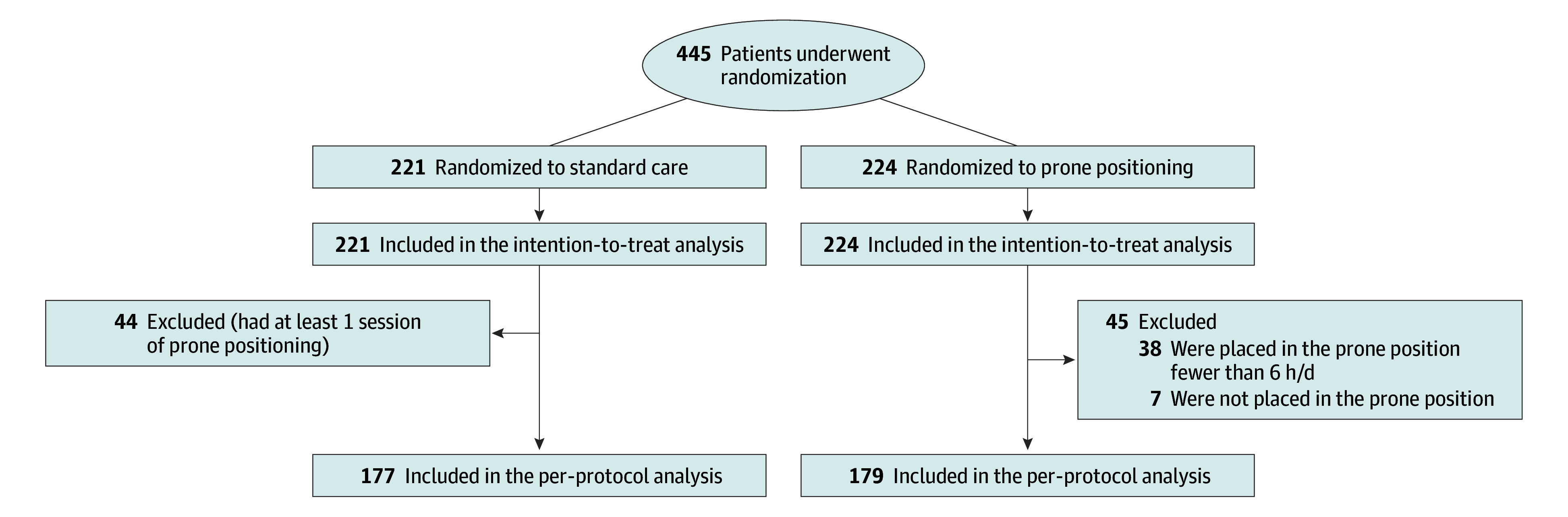
Flowchart Showing Enrollment, Randomization, and Inclusion in the Analysis

Eligible patients were randomly assigned in a 1:1 ratio to either APP or standard care (Figure 1). Randomization was stratified on study centers. The nature of the study intervention precluded a blind design for patients as well as health care practitioners.

In the intervention group, APP was regularly proposed to target at least 6 hours of proning a day. APP sessions of 2 hours were encouraged by regularly monitoring patients’ position. In the standard care (control) group, there was no positioning constraint, and spontaneous APP was not discouraged. Mobilization, especially sitting position, was encouraged in both groups.

All management decisions other than patient proning were made by the treating physician according to standard practice. Following intubation, prone positioning was considered as standard of care.

### Outcomes and Sample Size Calculation

The primary outcome was the need for intubation and/or incidence of death in the first 28 days of randomization.^5,12^ Prespecified secondary outcomes at 28 days of enrollment were days alive outside the intensive care unit (ICU), days alive outside the hospital, proportion of patients admitted to ICU (for patients not in ICU at baseline), and days alive and free from mechanical ventilation.

The PROVID trial was designed in a bayesian framework for which there was no definitive sample size determination before the start of the study that can be stopped according to stopping rules, as defined in the next section. However, we considered that a maximal limit of the sample size could be 250 patients per group based on a sample size for a frequentist approach that would allow an 80% power to detect an absolute difference of 10% between the 2 groups (ie, 15% vs 25% for the primary end point, corresponding to an OR of 0.53).

### Statistical Analysis

We specified prior distributions for both binary and continuous outcomes in the bayesian model. For binary outcomes, we used 5 prior distributions: 1 noninformative, corresponding to a distribution (normal [0-10]) for log (OR); 2 skeptical, centered at no effect with different strengths (moderate: normal [0-0.355]; strong: normal [0-0.205]) following Zampieri et al^13^; and 2 optimistic, centered at the expected effect (moderate: normal [−0.693 to 0.669], allowing 15% probability of harm; strong: normal [−0.693 to 0.421], allowing 5% probability of harm). For continuous outcomes with an expected effect of 2 days, we maintained the same framework with the same noninformative and skeptical prior distributions, while adjusting the optimistic prior distributions to be centered at 2 days (moderate: normal [2-1.93]; strong: normal [2-1.22], maintaining the same probabilities of harm as their binary counterparts).

We used Markov chains with Monte Carlo integrations to estimate posterior distribution for the estimations of statistical distributions of parameters, differences of interest, and ORs. With those distributions, the posterior probability of a more than trivial benefit (OR <0.95) and a more than trivial harm (OR >1.05) was calculated. For quantitative outcomes, the posterior probabilities for a benefit and a harm corresponding to 1 or 2 days’ difference in the APP group were also calculated. This approach contrasts with traditional point estimates, providing insights into the complete distribution of the relative risk and allows a more clinically meaningful interpretation of bayesian analyses’ results. For each analysis, 4 Markov chains with Monte Carlo integrations were used, each with 100 000 iterations and a burn-in period of 5000 iterations, to generate the posterior distribution. Convergence diagnostics, including trace plots and the Gelman-Rubin statistic, confirmed proper mixing and convergence across all parameters.

Early stop was defined based on evidence of non-null effect with a posterior probability of the OR greater than 1 being larger than 0.95 but also the posterior probability of a larger positive effect of prone positioning, defined as OR greater than 1.05 being larger than 0.8. Early stop was also possible in case of evidence of inefficacy OR less than 1 or deleterious effect of prone positioning, with threshold posterior probability fixed at 0.8 and 0.75, respectively.

Regarding the main efficacy criteria, posterior probability distributions are provided both graphically and by tables, showing posterior probabilities corresponding to different threshold values of the criterion. In addition, posterior probabilities of different threshold values of absolute relative risk reduction (RRR) were calculated, and different sensitivity analyses were performed using different prior distributions to evaluate how much the study conclusions depend on the a priori degree of skepticism on the effect of APP. The noninformative prior distributions were used for the primary analysis, while informative prior distributions were used for sensitivity analyses.

We performed a per-protocol analysis that included the patients in the standard care group who had never been in prone position during the study period and patients in the APP group who had been at least in prone position for 6 hours in 1 day during the study period. Intention-to-treat statistical analyses were performed from September to December 2024 using the brms package in R, version 4.3.2 (R Project for Statistical Computing). On August 26, 2021, the study steering committee discussed the results of a meta-trial^5^ and decided to stop the trial by considering that the strategy in the standard group had become unethical.

## Results

From July 2020 to August 2021, 445 patients (mean [SD] age, 60 [11] years; 116 females [26%] and 329 males [74%]) were enrolled in the PROVID trial. Most patients (429 [96%]) were recruited in France, and 16 patients (4%) were recruited in Mexico. Of the 445 patients, 224 (50%) were randomly assigned to the APP group and 221 (50%) to the standard care group (Figure 1). The study groups were well-matched at baseline (Table 1).

**Table 1.** Baseline Characteristics of Intention-to-Treat Population

Characteristic	Patients, No. (%)	
	Standard care group (n = 221)	APP group (n = 224)
Age, mean (SD), y	60 (11)	59 (12)
Sex		
Female	59 (27)	57 (25)
Male	162 (73)	167 (75)
Weight, mean (SD), kg	86 (20)	89 (20)
Height, mean (SD), cm	171 (9)	171 (10)
BMI, mean (SD)	29.2 (5.7)	30.2 (6.0)
Comorbidities		
Respiratory	44 (20)	46 (21)
Hypertension	102 (46)	86 (38)
Diabetes	58 (26)	57 (26)
Cancer or hemopathy	8 (4)	7 (3)
Liver disease	4 (2)	0 (0)
Physiological values at enrollment		
SBP, mean (SD), mm Hg	127 (19)	127 (20)
DBP, mean (SD), mm Hg	74 (13)	76 (13)
Heart rate, mean (SD), bpm	86 (17)	85 (17)
Respiratory rate, median (IQR), breaths/min	25 (22-30)	25 (20-30)
SpO_2_, mean (SD), %	94 (5)	94 (3)
SpO_2_ to FIO_2_ ratio, median (IQR)	150 (114-194)	155 (109-221)
Time to enrollment, median (IQR), d		
Time from symptoms to admission	8 (6-9)	7 (5-10)
Time from admission to randomization	1 (1-3)	1 (1-2)
Oxygenation support		
Low flow	63 (29)	73 (33)
High flow	147 (67)	145 (65)
NIV	11 (5)	6 (3)
Laboratory values at baseline, median (IQR)		
Creatinine, mg/dL	0.84 (0.71-1.06)	0.83 (0.68-1.06)
WBC count/μL	7020 (5020-9385)	6810 (5245-9408)
D-dimer, μg/mL	0.93 (0.57-1.59)	0.95 (0.62-1.42)
AST, U/L	57 (41-76)	57 (41-85)
ALT, U/L	41 (29-66)	40 (27-71)
Admission status at inclusion		
ICU	158 (71)	162 (72)
Ward	63 (29)	62 (28)

Abbreviations: ALT, alanine aminotransferase; APP, awake prone positioning; AST, aspartate aminotransferase; BMI, body mass index (calculated as weight in kilograms divided by height in meters squared); bpm, beats per minute; DBP, diastolic blood pressure; FIO_2_, fraction of inspired oxygen; ICU, intensive care unit; NIV, noninvasive ventilation; SBP, systolic blood pressure; SpO_2_, peripheral oxygen saturation; WBC, white blood cell.

SI conversion factors: To convert ALT and AST to microkatal per liter, multiply by 0.0167; creatinine to micromoles per liter, multiply by 88.4; D-dimer to nanomole per liter, multiply by 5.476; WBC to ×10^9^/L, multiply by 0.001.

At the time of randomization, 158 patients (71%) in the standard care group and 162 patients (72%) in the APP group were admitted to the ICU. The median (IQR) SpO_2_ to fraction of inspired oxygen (FIO_2_) ratio was 150 (114-194) and 155 (109-221) in the standard care and APP groups, respectively. Patients included from the ICU had a median (IQR) SpO_2_ to FIO_2_ ratio of 139 (100-178), while those included from the ward had a median (IQR) SpO_2_ to FIO_2_ ratio of 244 (165-285). At randomization, 292 patients (66%) were oxygenated through high-flow nasal canula (HFNC). The use of immunomodulators or antiviral drugs was similar between groups (eTable 1 in Supplement 2).

### Adherence to Awake Prone Positioning

In the APP group, 7 of 224 patients (3%) were never proned because they did not tolerate the position. Forty-five patients (20%) did not succeed in spending 6 hours in APP at least 1 day during the study period. Median (IQR) time spent in APP was 22 (10-42) hours over the study period, with a median (IQR) daily APP duration of 6 (2-8) hours and a median (IQR) number of days with APP of 4 (2-6) days. Forty-four of 221 patients (20%) in the standard care group were proned because patients turned spontaneously or the physician in charge suggested the position to improve oxygenation. The median (IQR) duration of daily APP was 6 (3-7.5) hours in the 63 patients included from the ward and was 6 (2-8) hours for the 158 patients included from the ICU. The median (IQR) number of days with APP was 4 (2-6) days for patients in the ward and 4 (3.5-6) days for patients in the ICU.

### Primary Outcome

**Figure 2.**
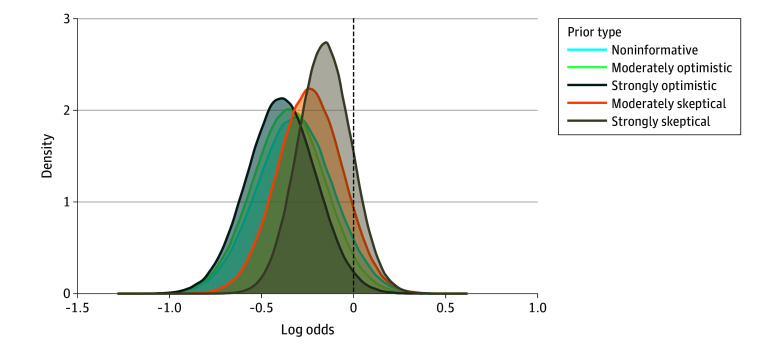
Posterior Probability Distribution for Odds Ratio of Intubation and/or Death for 5 Prior Distributions in the Intention-to-Treat Population

Using a noninformative prior distribution, the posterior probability that APP decreased intubation and/or death over the first 28 days of admission compared with standard care, was 93.8% (mean OR, 0.74; 95% credible interval [CrI], 0.48-1.09). The posterior median absolute RRR was 0.07 (95% CrI, 0.00-0.15) (Table 2). The posterior probability of a more than trivial benefit and a more than trivial harm in the APP group was 90.2% and 3.8%, respectively. Posterior probabilities for intubation and/or death for a range of 5 prior distributions are reported in eTable 2 in Supplement 2. With a moderately optimistic and a strongly optimistic prior distribution, the probability of OR less than 1 was 96.2% (mean OR, 0.72; 95% CrI, 0.48-1.04) and 98.2% (mean OR, 0.69; 95% CrI, 0.47-0.97), respectively. Under a moderately skeptical and a strongly skeptical prior distribution, the probability of OR less than 1 was 90.8% (mean OR, 0.80; 95% CrI, 0.56-1.12) and 86.1% (mean OR, 0.86; 95% CrI, 0.64-1.14), respectively (eTable 2 in Supplement 2). Figure 2 presents the posterior probability distribution for OR for each of the 5 prior distributions.

**Table 2.** Effect of Awake Prone Positioning Estimated by Bayesian Analysis According to a Noninformative Prior Distribution About Primary and Secondary Outcomes in the Intention-to-Treat Population

Outcome	Standard care group, No. (%) (n = 221)	APP group, No. (%) (n = 224)	Mean estimated difference (95% CrI)	Mean OR (95% CrI)	Posterior median absolute RRR (95% CrI)	Posterior probability of OR, %	Posterior probability of difference reduction, %
Primary							
Intubation and/or death within 28 d of enrollment	75 (34)	61 (27)	NA	0.74 (0.48 to 1.09)	0.07 (0.00 to 0.15)	93.8	NA
Intubation	67 (30)	55 (25)	NA	0.76 (0.49 to 1.13)	0.06 (0.00 to 0.14)	91.4	NA
Death	32 (15)	26 (12)	NA	0.78 (0.43 to 1.29)	0.04 (0.00 to 0.10)	85.1	NA
Secondary							
Days alive and free from mechanical ventilation within 28 d of enrollment, median (IQR)	28 (23 to 28)	28 (28 to 28)	0.33 (−1.37 to 2.03)	NA	NA	NA	64.9
Days alive outside the ICU within 28 d of enrollment, median (IQR)	21 (0 to 25)	22 (2.5 to 25)	1.28 (−0.78 to 3.34)	NA	NA	NA	88.8
Days alive outside the hospital within 28 d of enrollment, median (IQR)	13 (0 to 20)	16 (0 to 21)	1.55 (−0.22 to 3.32)	NA	NA	NA	95.7
ICU admission	16 (25)^a^	15 (24)^a^	NA	0.99 (0.44 to 1.91)	0.02 (0.00 to 0.06)	58.9	NA

Abbreviations: APP, awake prone positioning; CrI, credible interval; ICU, intensive care unit; NA, not applicable; OR, odds ratio; RRR, relative risk reduction.

^a^ From 63 patients in the standard care group and 62 patients in the APP group.

By day 28, 75 patients (34%) in the standard care group and 61 patients (27%) in the APP group had been intubated or died; 67 patients (30%) in the standard care group vs 55 patients (25%) in the APP group had been intubated; and 32 patients (15%) in the standard care group and 26 patients (12%) in the APP group had died (eFigure in Supplement 2). Death occurred in 25 (37%) of 67 mechanically ventilated patients in the standard care group compared with 20 (36%) of 55 mechanically ventilated patients in the APP group. Six (3%) and 8 (4%) patients in the APP group and standard care group, respectively, died without being intubated.

### Secondary Outcomes

Secondary outcomes are shown in Table 2. Using a noninformative prior distribution, the mean difference in the number of days alive and free from mechanical ventilation within 28 days between the APP group and the standard care group was 0.33 (95% CrI, −1.37 to 2.03) days. The mean difference in the number of days alive outside the ICU over the first 28 days of admission between the APP group and the standard care group was 1.28 (95% CrI, −0.78 to 3.34) days. The mean difference in the number of days alive outside the hospital over the first 28 days of admission between the APP group and the standard care group was 1.55 (95% CrI, −0.22 to 3.32) days. Probabilities corresponding to different prior distributions for secondary outcomes are shown in eTables 3 to 6 in Supplement 2.

### Per-Protocol Analysis

A total of 177 patients in the standard care group and 179 patients in the APP group fulfilled the criteria for the per-protocol analysis (Figure 1). In the APP group, the median (IQR) daily time in prone position was 6 (4-8) hours, for a total median (IQR) time of 28.5 (16.7-44.9) hours.

Primary and secondary outcomes in the per-protocol analysis are reported in Table 3. In these patients, the posterior probability that APP decreased intubation and/or death over the first 28 days of admission compared with standard care was 99.3% (mean OR, 0.58; 95% CrI, 0.35-0.89). The posterior median absolute RRR was 0.12 (95% CrI, 0.02-0.21). The posterior probability of a more than trivial benefit and a more than trivial harm in the APP group was 98.7% and 0.4%, respectively. Probabilities corresponding to different prior distributions are shown in eTable 7 in Supplement 2. By day 28, 60 of 177 patients (34%) in the standard care group and 40 of 179 patients (22%) in the APP group had been intubated or died. Probabilities corresponding to 5 prior distributions for secondary outcomes are provided in eTables 8 to 11 in Supplement 2.

**Table 3.** Effect of Awake Prone Positioning Estimated by Bayesian Analysis According to a Noninformative Prior Distribution About Primary and Secondary Outcomes in the Per-Protocol Population

Outcome	Standard care group, No. (%) (n = 177)	APP group, No. (%) (n = 179)	Mean estimated difference (95% CrI)	Mean OR (95% CrI)	Posterior median absolute RRR (95% CrI)	Posterior probability of RRR, %	Posterior probability of difference reduction, %
Primary							
Intubation and/or death within 28 d of enrollment	60 (34)	40 (22)	NA	0.58 (0.35 to 0.89)	0.12 (0.02 to 0.21)	99.3	NA
Intubation	53 (30)	37 (21)	NA	0.63 (0.37 to 0.98)	0.09 (0.01 to 0.18)	97.9	NA
Death	26 (15)	16 (9)	NA	0.60 (0.29 to 1.09)	0.06 (0.00 to 0.12)	95.6	NA
Secondary							
Days alive and free from mechanical ventilation within 28 d of enrollment, median (IQR)	28 (23.5 to 28)	28 (28 to 28)	0.77 (−1.07 to 2.61)	NA	NA	NA	79.4
Days alive outside the ICU within 28 d of enrollment, median (IQR)	21 (0 to 26)	22 (9.5 to 25)	1.40 (−0.85 to 3.64)	NA	NA	NA	88.9
Days alive outside the hospital within 28 d of enrollment, median (IQR)	15 (0 to 20)	16 (0 to 20)	1.40 (−0.57 to 3.36)	NA	NA	NA	91.9
ICU admission	13 (23)^a^	12 (26)^a^	NA	0.99 (0.40 to 2.06)	0.02 (0.00 to 0.06)	59.4	NA

Abbreviations: APP, awake prone positioning; CrI, credible interval; ICU, intensive care unit; NA, not applicable; OR, odds ratio; RRR, relative risk reduction.

^a^ Fifty-six patients in the standard care group and 47 patients in the APP group were not admitted into the ICU at the time of study enrollment.

### Subgroup Analysis

At the time of study enrollment, 320 patients were admitted to the ICU and 125 patients were admitted to the ward. Posterior probabilities for primary and secondary outcomes corresponding to different prior distributions are shown in eTables 12 and 13 in Supplement 2 for patients in ICUs and wards. We separated patients according to their body mass index (calculated as weight in kilograms divided by height in meters squared; <30 vs ≥30), and the posterior probabilities for primary and secondary outcomes corresponding to a noninformative prior distribution are reported in eTable 14 in Supplement 2.

## Discussion

In this RCT, APP showed a high probability of reduction in intubation and/or death over a wide range of prior distributions in patients with COVID-19–related respiratory failure who had no endotracheal intubation. In the beginning of the COVID-19 pandemic, APP showed an improvement in oxygenation and a reduction in work of breathing in patients with acute respiratory failure who were not intubated.^4,14^ Several RCTs have thus been conducted to evaluate APP for COVID-19 treatment in the general ward, in the ICU, or in mixed populations. In a meta-trial of 6 RCTs, Ehrmann et al^5^ reported a significant decrease in intubation rate but not mortality by day 28 in patients in the APP group among 1127 patients with oxygenotherapy delivered by HFNC. The mean (SD) SpO_2_ to FIO_2_ ratio was 148 (44) at inclusion, with a median (IQR) daily APP time of 5.0 (1.6-8.8) hours. The trial by Alhazzani et al^6^ included 400 patients in the ICU with acute respiratory failure due to COVID-19 infection; of these patients, 78% had oxygenation support by HFNC or noninvasive ventilation, with a median (IQR) SpO_2_ to FIO_2_ ratio of 132 (103-174). Alhazzani et al^6^ found no difference in intubation rate or mortality with APP, and the median (IQR) APP duration was 4.8 (1.8-8.0) hours per day. In the present study, patients had slightly less severe lung disease, with a median (IQR) SpO_2_ to FIO_2_ ratio of 150 (114-194); however, a meta-analysis of RCTs assessing APP in 3019 patients with COVID-19 and hypoxemic respiratory failure reported that patients with a SpO_2_ to FIO_2_ ratio between 155 to 232 were more likely to benefit from APP.^10^

We achieved daily APP time of 6 hours over a median (IQR) duration of 4 (2-6) days. This duration is greater than that reported in large RCTs.^5,6,15,16,17,18^ A duration of daily APP longer than 8 hours was associated with a significant decrease in intubation and/or mortality,^19,20^ and 10 hours daily of APP over the first 3 days were associated with improved survival.^10^ More recently, a clinician-driven strategy of applying APP in patients with COVID-19 infection and acute respiratory failure for a median (IQR) of 12 (11-14) hours a day significantly decreased intubation rate and mortality compared with a median (IQR) daily APP duration of 5 (2-8.9) hours, indicating that daily time in prone position is a key factor in the effect of APP.^21^ Although only 25% of patients achieved APP duration over 8 hours in our trial, we reported a high probability of reduction in endotracheal intubation and/or death. Thus, even with a daily exposure of less than 8 hours, APP provided benefit compared with those patients who were not proned.

In addition to improving oxygenation, APP was reported to increase inspiratory efforts,^2^ which may cause additional lung injury, raising the concern of worsening lung function by inappropriately delaying intubation. However, the mortality of intubated patients was similar in the APP and the standard care groups, which do not report evidence of harm with APP. One study^5^ found that APP improved oxygenation and reduced the respiratory rate in patients with COVID-19 and hypoxemic respiratory failure. Another study^22^ also showed APP improved lung aeration and led to substantial lung recruitment compared with staying in the supine position. These mechanisms could contribute to a decrease in intubation and/or death by improving lung function.

### Strengths and Limitations

Our study has several strengths. First, it has a large sample. Second, time in APP was homogeneously allocated across disease severity (ie, ward or ICU). Third, we used a bayesian approach to report results as posterior probabilities and CrIs, which are more easily interpretable for clinicians than *P* values or CIs as a measure of uncertainty of the treatment effect. The bayesian approach leads to the enrichment of the classical frequentist approach by providing a complete description of the treatment effect distribution, allowing results to be more interpretable for clinicians. It can assess the magnitude of the clinical effect. In this study, a conservative approach with a noninformative prior distribution showed a 93.8% posterior probability of decrease in death and/or intubation with APP. However, it was not unreasonable to think that there was a strongly enthusiastic prior distribution, which was associated in our study with a 98.2% probability of decrease in death and/or intubation. Prone positioning was already proven to be beneficial for intubated patients with severe respiratory failure,^1^ and early APP showed improvement in oxygenation in patients with COVID-19 and acute respiratory failure.^4^ Moreover, the cost of applying APP is low as it is easy to perform, exposes patients to few complications, and is rapidly reversible if not tolerated. Further studies are warranted to explore whether APP has beneficial outcomes in other settings of acute hypoxemic respiratory failure (ie, community-acquired pneumonia).

This RCT also has several limitations. First, the study was terminated before enrolling 500 patients. However, it is unlikely that inclusion of 55 additional patients would have changed the reported results. Second, we did not prevent patients from self-turning, and some patients in the standard care group were suggested to prone, which exposed part of that group to APP and may have led to understating the effect of APP, as illustrated by the per-protocol analysis showing greater differences than the intention-to-treat analysis. Third, by mixing patients from the ward and the ICU, the proportion of patients with severe hypoxemia was reduced. However, despite that, we reported a high probability of reduction in endotracheal intubation and/or death over a wide range of prior distributions. Fourth, we encouraged patient mobilization in both groups, especially sitting, which was associated with improved oxygenation in patients with COVID-19 developing hypoxemic respiratory failure.^23^ Mobilization may have curbed the effects of APP compared with standard care; however, we aimed to provide the best standard of care beside APP. Fifth, by removing patients who were not able to prone in the per-protocol analysis, we may have introduced a bias by selecting a low-risk group (ie, those who were able to prone). Thus, the results of the per-protocol analysis should be interpreted cautiously. Sixth, the blinding of the study intervention could not be achieved; clinicians could not be prevented from knowing patients’ prone or supine status. This fact may have affected the decision of intubation between groups.

## Conclusions

In the PROVID RCT involving nonintubated patients with COVID-19 and acute hypoxemic respiratory failure, daily APP for 6 hours compared with standard care showed a high probability of reduced endotracheal intubation and/or death at 28 days after enrollment over a wide range of prior distributions in the intention-to-treat analysis. Given the low cost and low risk for adverse event associated with APP, these results support its use in patients with hypoxemic respiratory failure due to COVID-19 infection.

## Notes

Supplement 1.
Trial Protocol

Supplement 2. **eTable 1.** Use of Immunomodulators and Antiviral Drugs in the Intention-to-Treat Population **eTable 2.** Probability of Treatment Effects Estimated by Bayesian Analysis According to Different Prior Beliefs About 'Intubation and/or Death' Benefit From Prone Positioning in Patients in the Intention-to-Treat Population **eTable 3.** Probability of Treatment Effects Estimated by Bayesian Analysis According to Different Prior Beliefs About 'Days Alive and Free From Mechanical Ventilation' Benefit From Prone Positioning in the Intention-to-Treat Population **eTable 4.** Probability of Treatment Effects Estimated by Bayesian Analysis According to Different Prior Beliefs About 'Days Alive Outside ICU' Benefit From Prone Positioning in the Intention-to-Treat Population **eTable 5.** Probability of Treatment Effects Estimated by Bayesian Analysis According to Different Prior Beliefs About 'Days Alive Outside the Hospital' Benefit From Prone Positioning in the Intention-to-Treat Population **eTable 6.** Probability of Treatment Effects Estimated by Bayesian Analysis According to Different Prior Beliefs About 'ICU Admission' Benefit From Prone Positioning in the Intention-to-Treat Population **eTable 7.** Probability of Treatment Effects Estimated by Bayesian Analysis According to Different Prior Beliefs About 'Intubation and/or Death' Benefit From Prone Positioning in the Per Protocol Population **eTable 8.** Probability of Treatment Effects Estimated by Bayesian Analysis According to Different Prior Beliefs About 'Days Alive and Free From Mechanical Ventilation' Benefit From Prone Positioning in the Per Protocol Population **eTable 9.** Probability of Treatment Effects Estimated by Bayesian Analysis According to Different Prior Beliefs About 'Days Alive Outside ICU' Benefit From Prone Positioning in the Per Protocol Population **eTable 10.** Probability of Treatment Effects Estimated by Bayesian Analysis According to Different Prior Beliefs About 'Days Alive Outside the Hospital' Benefit From Prone Positioning in the Per Protocol Population **eTable 11.** Probability of Treatment Effects Estimated by Bayesian Analysis According to Different Prior Beliefs About 'ICU Admission' Benefit From Prone Positioning in the Per Protocol Population **eTable 12.** Probability of Treatment Effects Estimated by Bayesian Analysis According to Different Prior Beliefs About 'Intubation and/or Death' Benefit From Prone Positioning in the Subgroup of Patients Admitted in ICU at the Time of Enrolment **eTable 13.** Probability of Treatment Effects Estimated by Bayesian Analysis According to Different Prior Beliefs About 'Intubation and/or Death' Benefit From Prone Positioning in the Subgroup of Patients Admitted in the Ward (Not in ICU) at the Time of Enrolment **eTable 14.** Probability of Treatment Effects Estimated by Bayesian Analysis According to a Non-informative Prior About 'Intubation and/or Death' Benefit From Prone Positioning in Patients According to their BMI **eFigure.** Kaplan Meier Survival Curve for A/ Tracheal Intubation and/or Death B/ Tracheal Intubation, C/ Death Within 28 Days of Enrollment in the Intention-to-Treat Population

Supplement 3.
PROVID Study Group

Supplement 4.
Data Sharing Statement
