# Supplementary material for: Awake Prone Positioning in Patients With COVID-19 Respiratory Failure: A Randomized Clinical Trial
Source: JAMA Netw Open. 2025 Dec 10;8(12):e2548201. doi: 10.1001/jamanetworkopen.2025.48201 (PMC12696593; doi:10.1001/jamanetworkopen.2025.48201)
Supplement: Supplement 1. — Trial Protocol [file jamanetwopen-e2548201-s001.pdf]

" PRONE POSITIONING IN COVID-19 OXYGENO-DEPENDENT PATIENTS IN SPONTANEOUS VENTILATION (PROVID STUDY)"

RESEARCH PROTOCOL INVOLVING THE HUMAN PERSON WITH MINIMAL RISKS AND CONSTRAINTS

Version N°01 dated 22/04/2020
Project code: APHP200504 / IDRCB N°: 2020-A01132-37

Coordinating investigator: Dr Anatole HARROIS
Anesthesia – Critical Care Bicêtre Hospital
78, rue du général Leclerc 94275 Le Kremlin Bicêtre
Tel: 01 45 21 34 41
Fax: 01 45 21 28 75
Email: anatole.harrois@aphp.fr

Scientific Manager: Prof. Jacques DURANTEAU
Anesthesia – Critical Care Bicêtre Hospital
78, rue du général Leclerc 94275 Le Kremlin Bicêtre
Tel: 01 45 21 34 41
Fax: 01 45 21 28 75
Email : jacques.duranteau@aphp.fr

Promoter: Assistance Publique – Hôpitaux de Paris (AP-HP)
And by delegation: Clinical Research and Innovation Department (DRCI)
Saint-Louis Hospital
1, avenue Claude Vellefaux
DRCI-Headquarters project referent: Elodie SOLER
Phone : 01 44 84 17 35
Email: elodie.soler@aphp.fr

Structure ensuring research Follow-up: Clinical Research Unit (CRU)
Prof. Éric VICAUT
200, rue du faubourg Saint Denis – 75010 PARIS
DRCI-URC project referent: Valérie MAZUR
Phone : 01 40 05 49 85
Email: valerie.mazur@aphp.fr

Clinical Research and Innovation Department (DRCI)
Hôpital Saint Louis 75010 PARIS

This document is the property of the DRCI/APHP. Any reproduction is strictly prohibited.
Version n°4.0 dated 31/05/2019

SIGNATURE PAGE OF A RESEARCH PROTOCOL

Research Code: APHP200504 / IDRCB N°: 2020-A01132-37

Title: *EFFECTS OF PRONE POSITIONING IN SPONTANEOUSLY VENTILATED PATIENTS WITH COVID19 HYPOXEMIC LUNG DISEASE* / PROVID

Version N° 1.0 dated 22/04/2020

The research will be conducted in accordance with the protocol, the best practices in force and the legislative and regulatory provisions in force.

The coordinating investigator:
Dr. Anatole HARROIS
Anaesthesia - Resuscitation Bicêtre Hospital
78, rue du général Leclerc
94275 Le Kremlin Bicêtre

Date: 22/04/2020
Signature:

The Proponent
Assistance Publique – Hôpitaux de Paris
Clinical Research and Innovation Department (DRCI)
Saint Louis Hospital
1 avenue Claude Vellefaux
75010 PARIS

Date: 22/04/2020
Signature:

Unité de Recherche Clinique
Pr E. VICAUT
Hôpital Fernand Widal
200, rue du Fb Saint-Denis 75010 Paris
Tél : 01 40 05 49 73

The research received approval from the CPP (Ethics committee) Sud Est II on 24/04/2020.

This document is the property of the DRCI/APHP. Any reproduction is strictly prohibited.
Version n°4.0 dated 31/05/2019

TABLE OF CONTENTS

1 SYNOPTIC SUMMARY 5

2 SCIENTIFIC RESEARCH JUSTIFICATION 8

2.1 CURRENT STATE OF KNOWLEDGE WITH REGARD TO RESEARCH 8
2.2 RESEARCH HYPOTHESIS 8
2.3 DESCRIPTION OF THE POPULATION TO BE STUDIED AND JUSTIFICATION FOR ITS CHOICE 8
2.4 DESCRIPTION OF THE ACTS PERFORMED OR PRODUCTS USED IN ACCORDANCE WITH THEIR CURRENT USE 9
2.5 DESCRIPTION OF ACTS AND PROCEDURES ADDED BY THE SEARCH 9
2.6 SUMMARY OF FORESEEABLE AND KNOWN BENEFITS AND RISKS FOR RESEARCH PARTICIPANTS 9

3 RESEARCH OBJECTIVES 9

3.1 MAIN OBJECTIVE OF THE RESEARCH 9
3.2 SECONDARY RESEARCH OBJECTIVES 10

4 RESEARCH DESIGN 10

4.1 PRIMARY ENDPOINT 10
4.2 SECONDARY ENDPOINTS 10

5 DESCRIPTION OF THE RESEARCH METHODOLOGY 10

5.1 EXPERIMENTAL DESIGN 10
5.2 NUMBER OF PARTICIPATING CENTRES (SEE LIST IN ADDENDUM) 11
5.3 DESCRIPTION OF MEASURES TAKEN TO REDUCE AND AVOID BIAS 11

6 CONDUCT OF THE RESEARCH 11

6.1 RESEARCH TIMELINE 12
6.1 SUMMARY TABLE OR DIAGRAM OF THE CHRONOLOGY OF THE SEARCH 13
6.2 DISTINCTION BETWEEN CARE AND RESEARCH 13

7 SELECTION CRITERIA 14

7.1 INCLUSION CRITERIA 14
7.2 NON-INCLUSION CRITERIA 14
7.3 RECRUITMENT PROCEDURES 14

8 STOP RULES 14

8.1 CRITERIA AND MODALITIES FOR PREMATURE TERMINATION OF ACTS/PROCEDURES/STRATEGIES PRACTICED IN THE CONTEXT OF RESEARCH 14
8.2 CRITERIA AND MODALITIES FOR PREMATURE TERMINATION OF PARTICIPATION IN A SUBJECT'S RESEARCH 15

9 EVALUATION OF EFFECTIVENESS 15

9.1 DESCRIPTION OF EFFICACY ENDPOINTS 15
9.2 METHODS AND PLANNED TIMELINES FOR MEASURING, COLLECTING, AND ANALYZING EFFICACY ENDPOINTS 16

10 VIGILANCE 16

11 SPECIFIC RESEARCH COMMITTEES 16

STEERING COMMITTEE 16

12 DATA MANAGEMENT 16

12.1 DATA COLLECTION METHODS 17
12.2 IDENTIFICATION OF DATA COLLECTED DIRECTLY FROM FIUS THAT WILL BE CONSIDERED SOURCE DATA 17
12.3 RIGHT OF ACCESS TO SOURCE DATA AND DOCUMENTS 17

This document is the property of the DRCI/APHP. Any reproduction is strictly prohibited.

Version n°4.0 dated 31/05/2019

12.4 DATA PROCESSING AND RETENTION OF DOCUMENTS AND DATA 18
12.5 DATA OWNERSHIP 18

13 STATISTICAL ASPECTS 18

13.1 DESCRIPTION OF THE PLANNED STATISTICAL METHODS: 18
STATISTICAL CRITERIA FOR STOPPING THE SEARCH. 19
METHOD OF ACCOUNTING FOR MISSING, UNUSED OR INVALID DATA 19
MANAGE CHANGES TO THE INITIAL STRATEGY ANALYSIS PLAN. 20
POPULATION SELECTION 20

14 QUALITY CONTROL AND ASSURANCE 20

14.1 GENERAL ORGANIZATION 20
14.2 CASE REPORT BOOK 21
14.3 NON-CONFORMANCE MANAGEMENT 21
14.4 AUDIT 21
14.5 COMMITMENT OF RESPONSIBILITIES OF THE PRINCIPAL INVESTIGATOR 22

15 ETHICAL AND LEGAL ASPECTS 22

15.1 PROCEDURES FOR INFORMING AND OBTAINING THE CONSENT OF PERSONS PARTICIPATING IN THE RESEARCH 22
15.2 PROHIBITION ON PARTICIPATION IN ANY OTHER RESEARCH OR EXCLUSION PERIOD PROVIDED FOR AT THE END OF THE RESEARCH, IF APPLICABLE 23
15.3 LEGAL OBLIGATIONS 23
15.4 REQUEST FOR AN OPINION FROM THE COMMITTEE FOR THE PROTECTION OF PERSONS CPP 23
15.5 INFORMATION FROM THE ANSM 23
15.6 PROCEDURES RELATING TO THE DATA PROTECTION REGULATIONS 23
15.7 RESEARCH CHANGES 24
15.8 FINAL RESEARCH REPORT 24
15.9 ARCHIVING 24

16 FINANCING AND INSURANCE 24

16.1 SOURCE OF FUNDING 24
16.2 INSURANCE 25

17 PUBLICATION RULES 25

17.1 MENTION OF THE AP-HP'S AFFILIATION FOR PROJECTS PROMOTED BY THE AP-HP 25
17.2 MENTION OF THE AP-HP PROMOTER (DRCI) IN THE MANUSCRIPT'S ACKNOWLEDGMENTS 25
17.3 MENTION OF THE FUNDER IN THE MANUSCRIPT'S ACKNOWLEDGMENTS 25

18 BIBLIOGRAPHY 25

19 LIST OF PROTOCOL ADDENDA 27

19.1 LIST OF INVESTIGATORS ADDENDUM NUMBER 1 27
19.2 PATIENT COLLECTION FORM ADDENDUM NUMBER 2 28

This document is the property of the DRCI/APHP. Any reproduction is strictly prohibited.

Version n°4.0 dated 31/05/2019

1 SYNOPTIC SUMMARY

Full title	Effects of prone positioning in spontaneous breathing patients with COVID-19 respiratory failure
Acronym/Reference	PROVID
Coordinating Investigator	Dr HARROIS Anatole
Scientific Manager	Prof. DURANTEAU Jacques
Promoter	Assistance Publique – Hôpitaux de Paris
Scientific justification	The current COVID-19 epidemic is responsible for a significant number of severe hypoxemic respiratory distress that initially requires oxygen therapy (up to 15 L/min). This respiratory distress progresses to adult respiratory distress syndrome (ARDS) with the need to introduce mechanical ventilation treatment in intensive care. During ARDS, mechanical ventilation is based on the application of small tidal volumes (6 ml/kg), titrated end-tidal pressure to achieve the best pulmonary compliance without exceeding a plateau pressure of 30 cm H_2O and daily prone position (PP) placement when the P/F < 150 and the response to PP in terms of oxygenation is satisfactory. Prone positioning showed a benefit in terms of survival in non-COVID ARDS, linked to a better distribution of pulmonary ventilation. However, despite this optimized management of ARDS including PP, Chinese intensive care colleagues report a mortality of around 50% of COVID ARDS treated in the intensive care unit (ICU). Before admission to the ICU, patients with COVID pneumonitis go through different stages of oxygen repetition but we observed that oxygen requirement of 3L/min in the acute phase of the disease is strongly associated with the occurrence of severe respiratory distress.<br>We hypothesize that prone positioning patients breathing spontaneously (=not intubated) from the oxygen request stage of 3L/min would prevent the respiratory deterioration of COVID patients. This early stage PP is easy to perform because patients can mobilize themselves to make the position change. In addition, prevention of severe respiratory distress is a major objective during an epidemic during which resources in intensive care beds are limited.
Objective and primary endpoint	**Objective**: To show that daily prone positioning (objective of at least 6 hours a day) in COVID-19 patients with respiratory failure requiring oxygen therapy of at least 3L/min to obtain a saturation ≥ 95% reduces the proportion of patients requiring intubation or the occurrence of death.<br>**Composite Criterion**: Meeting one of the following criteria<br>- Use of intubation<br>- Death<br>Criteria assessed up to 28 days after inclusion
Secondary objectives and endpoints	**Secondary Objectives**:<br>To show that daily prone positioning (objective of at least 6 hours) in COVID-19 patients with respiratory involvement requiring oxygen therapy of at least 3L/min decreases:<br>- the proportion of patients admitted to intensive care (among those who were in the conventional hospitalization department at baseline)<br>-the use of intubation<br>- the use of non-invasive ventilation or high-flow oxygen therapy (for those who were not already receiving it at baseline)<br>-the length of ICU stay

This document is the property of the DRCI/APHP. Any reproduction is strictly prohibited.
Version n°4.0 dated 31/05/2019

	- the length of hospital stay<br>- the maximum flow rate of oxygen therapy (nasal or mask)<br><br>**<u>Secondary Criteria</u>**:<br>- the proportion of patients admitted to intensive care (among those who were in the conventional hospitalization department at baseline)<br>-Number of days alive without invasive ventilation in the first 28 days<br>- Number of days alive out of intensive care in the first 28 days<br>- Number of days alive out of hospital in the first 28 days<br>- Number of days alive without NIV or high-flow oxygen therapy in the first 28 days (for those who did not benefit at baseline)<br>- maximum flow rate of oxygen therapy over the first 28 days
Experimental design	Prospective, randomized, open-label, multicenter study (stratified on the patient's status in ICU/out of ICU).
Population concerned	Patients admitted into a COVID unit for respiratory failure, requiring oxygen therapy.
Inclusion criteria	-Adult COVID-19 patient (positive PCR or typical CT imaging), not intubated, requiring oxygen therapy at a rate of at least 3L/min to have a finger saturation greater than or equal to 95%<br>- Patient able to manage alone, intellectually and physically, prone positioning<br>-Absence of limitation of therapies (in particular no limitation on intubation)<br>-Patient affiliated to a social security scheme
Non-inclusion criteria	Patient aged > 80 years<br>Pregnant women<br>Inability to prone
Acts or Product being researched	Prone position for at least 6 hours daily with consecutive two-hour periods (if possible).
Comparator group	Patient without imposed positioning constraints
Other acts or procedures added by the search	No
Expected benefits for participants and society	We expect an improvement in respiratory care for patients, a reduction in the use of intensive care services and intubation, and consequently a saving in intensive care resources in times of epidemic
Minimal risks and constraints added by research	Placing patients in prone position with non-severe respiratory distress may induce discomfort.
Practical procedure	Patients who meet the criteria and are admitted to the COVID ward will be offered to participate in the study orally and then a written consent will be obtained. Notification of the patient's participation in this study will be listed in the patient's medical record.<br>Randomization will be done via cleanweb. A paper CRF will be completed in each centre and sent by photo or scan to the CRU for collection in the e-CRF. Following randomization, patients in the interventional group will be instructed to spend at least 6 hours on their stomach (PP). They will get into the position on their own and regularly note the time spent in PP. Patients in the control group will do as usual without external intervention on the positioning to be adopted. It will thus be possible to compare the expected results of the group in PP at least 6 h/24h to the group with standard behavior.
Number of Selected Subjects	At least 500 patients
Number of centres	This research will take place in 21 centres in France and Mexico
Research Timeline	Specify:<br>• Duration of inclusion: 18 months

This document is the property of the DRCI/APHP. Any reproduction is strictly prohibited.
Version n°4.0 dated 31/05/2019

	• duration of participation (treatment + follow-up): 28 days<br>• Total duration: 19 months<br>• Duration of prohibition from participation in other research and rationale: No prohibition from participating in other intervention research
Number of planned inclusions per centre per month	At least 4 patients per department per month and more if possible
Statistical analysis	This is a Bayesian study whose analysis will be based on the calculations of the a posteriori probabilities for a number of hypotheses. There is therefore no fixed sample size as in a frequentist analysis. The stopping rules will be based on the probability calculations conditional on the data of an OR>1, corresponding to a non-zero effect, but also of an OR>1.05 corresponding to a more marked effect, but also on the probabilities of OR<1 ineffectiveness or of a marked deleterious effect OR < 0.95 with respective probability thresholds set at 0.95,0.80,0.8,0.75. Vague priors but adapted to Bayesian studies using ORs will be used. The maximum size of the study is set at N=500 patients (based on the size of a frequentist study with a power of 80% to demonstrate a difference of 10% between the 2 groups (i.e. 25% vs 15%). The simulations carried out show a high probability that the study will be conclusive after the inclusion of less than 300 patients.
Source of funding	*Submission to the PHRC (Ministry of Health and Solidarity) COVID*

This document is the property of the DRCI/APHP. Any reproduction is strictly prohibited.
Version n°4.0 dated 31/05/2019

2 SCIENTIFIC RESEARCH JUSTIFICATION

2.1 Current state of knowledge with regard to research

2.1.1 *On the pathology*

The current COVID-19 epidemic is responsible for a significant number of severe hypoxemic respiratory distress that initially requires oxygen therapy (up to 15 L/min). This respiratory distress progresses to adult respiratory distress syndrome (ARDS) with the need for mechanical ventilation in intensive care. During ARDS, mechanical ventilation is based on the application of small tidal volumes (6 ml/kg), titrated end-tidal pressure to achieve the best pulmonary compliance without exceeding a plateau pressure of 30 cm H_2O and daily prone positioning (PP) when the P/F ratio < 150. Prone positioning showed a survival benefit in non-COVID ARDS, linked to a better distribution of pulmonary ventilation[1]. However, despite this optimized management of ARDS including PP, Chinese intensive care colleagues report a mortality of around 50% of COVID ARDS treated in intensive care[2]. Before admission to the ICU, patients with COVID pneumonia go through different stages of oxygen requirements, but we observed that an oxygen requirement of 3L/min in the acute phase of the disease is strongly associated with the occurrence of severe respiratory distress.

2.1.2 *On the reference strategies/procedures*

The management of respiratory distress related to COVID-19 infection has just been the subject of two recommendations issued by the "surviving sepsis campain" and by the World Health Organization[3,4].
In patients breathing spontaneously, it is recommended to administer oxygen therapy to achieve a SpO_2 between 92 and 96%. If standard oxygen therapy is insufficient, it is recommended to use high-flow nasal oxygen therapy up to 60L/min rather than non-invasive ventilation. If the latter is insufficient, then intubation for mechanical ventilation is necessary. Administration of a tidal volume between 4 and 8 mL/kg and the use of a positive end expiratory pressure (8 to 14 cmH_2O) without exceeding a plateau pressure of 30 cmH_2O are recommended. Prone position is used when the PaO_2/FiO_2 ratio is less than 150 in mechanically ventilated patients.
There is no recommendation on the positioning of patients on spontaneous ventilation. Usually, in the ward or in intensive care, patients on oxygen therapy for pneumonia remain in the supine position. Daily prone positioning could improve pulmonary ventilation in patients on spontaneous ventilation and thus prevent intubation.

2.2 Research hypothesis

We hypothesize that the prone position of patients in spontaneous ventilation (=not intubated) from the oxygen requirement stage of 3L/min prevents respiratory deterioration in COVID-19 patients. This early stage PP is easy to perform because patients can mobilize themselves to make the change of position. In addition, prevention of some of the severe respiratory distress is a major objective during an epidemic during which resources in intensive care beds are limited.

2.3 Description of the population to be studied and justification for its choice

Adult COVID-19 patients (PCR or typical CT imaging), not intubated, requiring oxygen therapy at a rate of at least 3L/min to get a peripheral saturation greater than or equal to 95%.
We observed during the recent management of patients in the context of the current COVID epidemic that oxygen supply at more than 3L/min was associated with a high proportion of deterioration with severe respiratory distress.

This document is the property of the DRCI/APHP. Any reproduction is strictly prohibited.
Version n°4.0 dated 31/05/2019

Thus, among patients admitted to hospital in China for COVID-19 pneumonia, 26% are secondarily admitted to intensive care, among which 50% of patients require mechanical ventilation [5–7]. However, only 80% of patients were oxygen-requiring in the Guan et al. series. Including patients requiring at least 3L/min of oxygen, some of which will be included in the intensive care unit and already on high-flow oxygen therapy, we estimate that 20% of the patients included will require mechanical ventilation or die in the arm without prone positioning.

2.4 Description of the acts performed or products used in accordance with their current use

Patients receive oxygen therapy through a mask, or even high-flow nasal oxygen therapy or non invasive ventilation to achieve an SpO_2 between 96% and above. Patients are usually placed in the supine position with the torso raised at 30°. An arterial catheter (radial or femoral) is placed in response to the need to take regular check-ups (blood gases in particular) to monitor the respiratory function of patients. A central venous catheter is placed when the patient requires sedation and/or catecholamine administration to maintain an average blood pressure level above 65 mmHg. In the case of intubation, the patient is intubated by the orotracheal route and is connected to a ventilator for the delivery of a tidal volume of 4 to 8 mL/kg, a PEEP of 8 to 14 mmHg without exceeding a plateau pressure of 30 cm of H_2O.

2.5 Description of acts and procedures added by the research

Prone position for at least 6 hours / 24 hours is added as well as the fact that the patient (or the nurse in charge) has to note alone the time spent in the position on a specific document provided by the research. Patients will lie on their stomach with their head turned to the side and a cushion placed under the chest at their convenience.
We do not want to overload caregivers, which is why we will only recruit patients who are completely autonomous. Caregivers will remind patients to remember to move to the prone position and note the time spent in this position only at times of care. No reminders outside of the field, so no additional contacts induced by the research.

2.6 Summary of foreseeable and known benefits and risks for research participants

By avoiding intubation of patients with COVID-19 hypoxemic respiratory failure thanks to prone positioning, we believe we can improve lung aeration, improve patient oxygenation, prevent intubation, and improve survival. The second expected benefit is the optimization of the management of ICU beds on a just-in-time basis during this pandemic period.
In some people, the prone position can induce discomfort, which, if not tolerated, can be divided at the discretion of the patient.

3 RESEARCH OBJECTIVES

3.1 Main objective of the research

To show that daily prone positioning (objective of at least 6 hours/day) in COVID-19 patients with respiratory failure requiring oxygen therapy of at least 3L/min to obtain a saturation ≥ 95% reduces the proportion of patients requiring intubation and/or death.

This document is the property of the DRCI/APHP. Any reproduction is strictly prohibited.
Version n°4.0 dated 31/05/2019

3.2 Secondary Research Objectives

To show that daily prone placement (objective of at least 6 hours) in COVID patients with respiratory involvement requiring oxygen therapy of at least 3L/min decreases:

– the proportion of patients admitted to intensive care (among those who were in the conventional hospitalization department at baseline)
– the use of intubation
– the use of non-invasive ventilation or high-flow oxygen therapy (for those who were not already receiving it at baseline)
– length of ICU stay
– length of hospital stay
– the maximum flow rate of oxygen therapy (nasal or mask)

4 RESEARCH DESIGN

4.1 Primary endpoint

Composite Outcome : Achievement of one of the following criteria (if absent at the time of randomization):

- Use of intubation
- Death

Criteria assessed up to 28 days after inclusion

4.2 Secondary endpoints

- The proportion of patients admitted to intensive care (among those who were in the conventional hospitalization department at baseline)
- Number of days alive without invasive ventilation in the first 28 days
- Number of days alive out of intensive care in the first 28 days
- Number of days alive out of hospital in the first 28 days
- Number of days alive without NIV or high-flow oxygen therapy in the first 28 days (for those who did not benefit at baseline)
- Maximum Flow Rate of Oxygen Therapy Over 28 Days

5 DESCRIPTION OF THE RESEARCH METHODOLOGY

5.1 Experimental design

Comparison of two positioning strategies to prevent respiratory complications related to COVID-19 respiratory infections

Randomized study, two comparison groups

Group A: interventional group: Patients will be instructed to spend at least 6 hours in the prone position (PP). They will put themselves in the position and regularly note (or the nurse in charge) the time spent in PP on a specific document provided by the research. Patients will lie on their stomach with their head turned to the side and a cushion placed under the chest at their convenience.

Group B: control group: Patients will not have any particular positioning instructions.

This document is the property of the DRCI/APHP. Any reproduction is strictly prohibited.
Version n°4.0 dated 31/05/2019

5.2 Number of participating centres (see list in addendum)

It is a multicenter research carried out in 12 hospitals in France.

- **Recruiting Centres**

Conventional hospitalization services dedicated to the care of COVID-19 patients will receive patients who meet the inclusion criteria (non-intubated patients requiring 3L/min of O_2 or more to obtain a peripheral saturation greater than 95%). The intensive care units will also include patients (either on simple oxygen therapy, on high-flow oxygen therapy, or on non-invasive ventilation) on these same criteria.

5.3 Description of measures taken to reduce and avoid bias

5.3.1 Subject Identification

Patients who will be offered to participate in the study should not be influenced by the assumption that the prone position would be more effective in improving their respiratory capacity compared to patients who will be given the choice of their positioning during medical management. This is why we have written an information note that makes sure not to influence patients who are not in the intervention group so that they keep the position of their choice without external interference in order to obtain results as relevant as possible.

5.3.2 In this research, the subjects will be identified as follows:

- Center No. (3 numerical positions) – I__I__I__I
- number of selection of the person in the centre (4 numerical positions) - I__I__I__I__I
- Initial last name - initial first name - I__I/I__I

This reference is unique and will be kept for the duration of the research.

5.3.3 Randomization

The patient randomization list will be drawn up according to a stratified randomization table by center and established by the F-Widal-Lariboisière clinical research unit.
Randomization will be done after verification of the inclusion and non-inclusion criteria, in the department where the patient has signed his consent (inclusion).
The investigator will perform randomization via Cleanweb software.
After getting to know the group, he will explain the strategy to the patient

Group A: interventional group: Patients will be instructed to spend at least 6 hours in the prone position (PP). They will put themselves in the position and regularly note the time spent in PP (or the nurse in charge) on a specific document provided by the research. Patients will lie on their stomach with their head turned to the side and a cushion placed under the chest at their convenience.

Group B: control group: Patients will not have any particular positioning instructions.

6 CONDUCT OF THE RESEARCH

This document is the property of the DRCI/APHP. Any reproduction is strictly prohibited.
Version n°4.0 dated 31/05/2019

Persons whose consent is sought	Who informs and collects the consent of the person	When the person is informed	When the consent of the person is obtained
The person who lends himself to the research	The principal investigator or collaborator who is a registered and research-trained physician (pulmonologists, internists, infectious disease specialists and intensive care specialists)	Selection visit as part of the treatment at the time of care.	After a reasonable period of reflection with the patient, knowing that the care of these patients will have to be rapid and the sooner the procedure is put in place, the more convincing the results will be.

6.1 Research Timeline

6.1.1 Inclusion visit.

For 18 months following the validation of the ethics committee, patients meeting the criteria and admitted to the COVID ward or intensive care in the 21 centers within the 11 hospitals in France and Mexico referenced for this study will be offered to participate in the study orally after verification of the inclusion and non-inclusion criteria and then a written consent will be collected. Notification of the patient's participation in this study will be listed in the patient's medical record.
We will collect:
Socio-demographic data: age, sex, weight, height, date of onset of symptoms, date of hospital admission
Constants: systolic blood pressure (SBP), diastolic blood pressure (DBP), heart rate (HR), body temperature, respiratory rate (RH), oxygen saturation, oxygen therapy flow.
Collection of the date of the last chest CT scan before inclusion with percentage of lung parenchyma involvement on CT images. Chest CT scan is not required for the study if the patient has not had one.
Biology: white blood cell counts, serum creatinine, D-Dimer, AST, and ALT (liver enzymes). This assessment will be collected, if it exists, made within the usual framework.
Medical history: chronic respiratory disease, cardiovascular disease, liver disease, diabetes and cancer or hematopathy under treatment.
Randomization will be done following the collection of written consent, via Cleanweb software.

6.1.2 Follow-up visits

From this point on, patients in the interventional group (Group A) will be instructed to spend at least 6 hours on their stomach (PP). They will put themselves in the position. They will stand on their stomach with their head turned to the side and a cushion placed under the chest at their convenience and will regularly note the time spent in PP. They (or the nurse in charge) will have at their disposal a collection sheet and a pen.
Patients in the control group (Group B) will do as usual without external intervention on the positioning to be adopted.

For a maximum of 28 days, a daily reading of the

- Vital status
- Transfer to intensive care for patients included in a ward unit

This document is the property of the DRCI/APHP. Any reproduction is strictly prohibited.
Version n°4.0 dated 31/05/2019

- Use of intubation
- Use of an alternative ventilation technique (NIV, high-flow nasal oxygen therapy
- Maximum Oxygen Therapy Flow Rate (Nasal or Mask)

At the end of the stay (maximum on the 28th day): we will collect
- Length of ICU stay
- Length of hospital stay

If the patient is discharged before day 28 (maximum duration of study participation)
- Your vital status will be searched for up to 2 months after discharge by phone call

The total duration of the study will be 19 months.

Length of Inclusion Period	18 months
Duration of participation of subjects	28 days
Search for vital status	Up to two months after discharge from hospital by phone call

6.1 Summary table or diagram of the chronology of the search

	Selection Day 1	Inclusion/ D1 Randomization	Visits D1 to D28 maximum	Up to two months after inclusion
Oral information	✓			
Collection of written consent	✓			
Socio-demographic data		✓		
Randomization		✓		
Positioning Explained		✓		
Reminder during care for the interventional group on the positioning to adopt at least 6 hours a day		✓	✓	✓
Constant<br>Oxygen therapy levels<br>Become<br>Vital status		✓	✓	
Oxygen Therapy Flow Rate	✓		✓	
Telephone call for vital status if patient discharged from hospital before 28 days				✓

6.2 Distinction between care and research

TABLE: Distinction between acts related to "care" and acts added by "research"

This document is the property of the DRCI/APHP. Any reproduction is strictly prohibited.
Version n°4.0 dated 31/05/2019

Research interventions	Care-related acts, procedures and treatments	Acts, procedures added by research
**Randomization**		Randomization will be applied to all patients
**Strategy**	Position left to the patient's discretion	Prone position at least 6 hours / 24 hours

7 SELECTION CRITERIA

7.1 Inclusion criteria

- Non-intubated adult COVID-19 patients (positive PCR or imaging: typical CT scan) requiring nasal oxygen therapy at a rate of at least 3 L/min to have a peripheral saturation greater than or equal to 95%
- Patient able to manage prone positioning on their own intellectually and physically
- Lack of limitation of therapies
- Patient affiliated to a social security scheme

7.2 Non-inclusion criteria

- Patient aged > 80 years
- Pregnant women
- Inability to prone

7.3 Recruitment procedures

- Patients will be recruited in the traditional inpatient ward (pulmonology, internal medicine, infectious disease) and in critical care.
- The epidemic is currently responsible for the hospitalization of 20 to 50 patients per day in the hospitals participating in the research. Half of them will require oxygen at more than 3L/min

	Number of Subjects
Total number of selected topics	500
Number of centres	21
Inclusion period (months)	18 months
Number of subjects / centre	24
**Number of topics / center / month**	4 or more

8 STOP RULES

8.1 Criteria and modalities for premature termination of acts/procedures/strategies practiced in the context of research

Different situations exist:

- Temporary discontinuation, the investigator should document the reason for the discontinuation and its resumption in the subject's source file and CRF

This document is the property of the DRCI/APHP. Any reproduction is strictly prohibited.
Version n°4.0 dated 31/05/2019

- Premature discontinuation, but subject remains in the research, until the end of their participation, the investigator should document the reason.

8.2 Criteria and modalities for premature termination of participation in a subject's research

- Any subject can stop participating in the research at any time and for any reason.
- The investigator may temporarily or permanently discontinue a subject's participation in research for any reason that impacts the safety of the subject or that would be in the best interests of the subject.

In the event of premature termination of a subject's research, or withdrawal of consent, the data concerning him or her collected before the premature termination may be used.

The report card must list the various reasons for stopping participation in research:

- ☐ Adverse effect
- ☐ Other medical problem
- ☐ Personal reason of the subject
- ☐ Explicit Withdrawal of Consent.

Monitoring methods and collection schedule for this data:

For the duration of the study (28 days) and up to two months after the patient's discharge from the hospital if discharged before the 28th day.

8.2.1 Arrangements for replacing these persons, if applicable

Not applicable.

8.2.2 Stopping part or all of the research

The promoter AP-HP reserves the right to permanently suspend inclusions, at any time, if it turns out that the inclusion objectives are not achieved.

9 EVALUATION OF EFFECTIVENESS

9.1 Description of efficacy endpoints

Composite Outcome : Achievement of one of the following criteria (if absent at the time of randomization):

- Use of intubation
- Death

Criteria assessed up to 28 days after inclusion

Secondary endpoints

- The proportion of patients admitted to intensive care (among those who were in the conventional hospitalization department at baseline)
- Number of days alive without invasive ventilation in the first 28 days
- Number of days alive out of intensive care in the first 28 days
- Number of days alive out of hospital in the first 28 days

This document is the property of the DRCI/APHP. Any reproduction is strictly prohibited.
Version n°4.0 dated 31/05/2019

- Number of days alive without NIV or high-flow oxygen therapy in the first 28 days (for those who did not benefit at baseline)
- Maximum rate of oxygen therapy over the course of 28 days.

9.2 Methods and planned timelines for measuring, collecting, and analyzing efficacy endpoints

The evaluation parameters are provided for in the case report form and will be collected by the designated investigators from the time of inclusion until the 28th day of participation. A telephone call may be made to the patient if he or she has been discharged from hospital before the 28th day in order to collect his or her vital status.
The data noted by the patient on the "time spent in PP" data collection sheet will be collected regularly in the paper CRF by the investigators.
The data collection in the e-CRF will be done by a TEC of the F-Widal-Lariboisière CRU, following the sending of the CRF pages by the centres by photos or scans with anonymised data.

10 VIGILANCE

In the context of this research mentioned in 2° of Article 1121-1 of the CSP, adverse events (serious or not) are not to be notified to the sponsor. The notification must be made within the framework of the vigilance put in place in the context of care for the practice that is the subject of the research.
Nevertheless, in accordance with Article R 1123-59 of the CSP, the promoter shall inform the competent authority and the Committee for the Protection of Persons without delay of the new facts defined in 12° of Article R. 1123-46 and, where applicable, of the measures taken.

11 SPECIFIC RESEARCH COMMITTEES

Steering Committee

The steering committee is made up of:

Coordinating investigator: Dr Anatole Harrois, Department of Anaesthesia and Critical Care at Bicêtre Hospital. Organizes research on the general plan and coordinates information.

Scientific director: Prof. Jacques Duranteau, Department of Anaesthesia and Critical Care at the Bicêtre Hospital. Also organizes research and coordinates information.

Biostatistician: Prof. Eric Vicaut Lariboisière-Fernand Widal Clinical Research Unit. Determines the methodology and monitors the progress of the research with the help of the team of the Lariboisière-Fernand Widal Clinical Research Unit.

The steering committee is in regular telephone contact for the organization of the research and also meets formally by videoconference on a regular basis.

12 DATA MANAGEMENT

This document is the property of the DRCI/APHP. Any reproduction is strictly prohibited.
Version n°4.0 dated 31/05/2019

12.1 Data collection methods

Initially, and in view of the current circumstances, the data will be collected on a paper observation notebook and then transmitted to the F-Widal-Lariboisière clinical resaerch unit by photos or scanners and transcribed on e-CRF by a TEC.

12.2 Identification of data collected directly from FIUs that will be considered source data

All data will be collected from the patient's medical record of all documents, including the patient collection form.

12.3 Right of access to source data and documents

12.3.1 ***Access to data***

In accordance with GCPs:

- the sponsor is responsible for obtaining the agreement of all parties involved in the research in order to ensure direct access to all research locations, source data, source documents and reports for the purpose of quality control and audit by the sponsor,
- The investigators will make available to the persons in charge of monitoring, quality control, auditing research involving human persons, the documents and individual data strictly necessary for this control, in accordance with the legislative and regulatory provisions in force

12.3.2 ***Source documents***

Source documents, being defined as any original document or object that proves the existence or accuracy of a data or fact recorded during the research, will be kept for 15 years by the investigator or by the hospital in the case of a hospital medical record.

In the context of this research, the source documents are the medical records of each department in which the patient will have passed at some point in his or her care. The medical file, as well as the nursing and nursing assistant care file, paper or computerized version. The group A patient collection form will also be part of the source documents.

12.3.3 ***Data Privacy***

The persons responsible for quality control of research involving human beings (Article L.1121-3 of the Public Health Code) will take all necessary precautions to ensure the confidentiality of information relating to the research, to the persons who take part in it and in particular with regard to their identity and the results obtained.
These persons, in the same way as the investigators themselves, are subject to professional secrecy (under the conditions defined by Articles 226-13 and 226-14 of the Criminal Code).
During research involving humans and at the end of it, the data collected on suitable persons and transmitted to the sponsor by the investigators (or any other specialized stakeholders) will be made non-identifying.
They must not under any circumstances display the names of the persons concerned or their addresses in plain text.

The sponsor will ensure that each person who participates in the research has given his or her written consent for access to the individual data concerning him or her that is strictly necessary for the quality control of the research.

This document is the property of the DRCI/APHP. Any reproduction is strictly prohibited.
Version n°4.0 dated 31/05/2019

12.4 Data processing and retention of documents and data

12.4.1 *Identification of the data controller and place of data processing*

The creation of the database, the data entry, and the methods of protecting the confidentiality and security of the data, will be carried out according to the quality procedures in force at the Clinical Research Unit (CRU) Lariboisière – Saint Louis, under the responsibility of Prof. Eric Vicaut.
The analyses will be carried out under the responsibility of Prof. Eric Vicaut using WINBUGS or R software.

The observations will be entered through a paper observation book. These CRFs will be entered by the investigator or a data entry technician, then transmitted to the CRU according to a predefined rhythm in the form of a photo or scanned and anonymised.
A TEC at the F-Widal CRU will transcribe it in the e-crf.
The database will be backed up on a daily basis. All of these operations will be carried out continuously, i.e. as the data is collected.
The computer control program for the consistency of information must be developed on the basis of a validation plan describing in detail the checks to be carried out for each variable (consistency of dates and deadlines, consistency of conditional variables, invalid values, bounds, missing data, compliance with criteria predefined in the protocol, etc.). "Inconsistent" data (not related to an input error) are edited once a month and sent to the TEC for verification of the information in the patient files.

12.4.2 *Data Entry*

Paper CRF : The data entry will be carried out by a TEC of the URC F-Widal-Lariboisière, Professor VICAUT by staff dedicated to this purpose on data collection notebooks with non-identifying data
E-CRF: The entry of data that has been made non-identifying will be carried out on the cleanweb via a web browser.

12.5 Data ownership

AP-HP is the owner of the data and no use or transmission to a third party may be carried out without its prior consent.

13 STATISTICAL ASPECTS

13.1 Description of the planned statistical methods:

Study size:

This is a Bayesian study whose analysis will be based on the calculations of the a posteriori probabilities for a number of hypotheses. There is therefore no fixed sample size as in a frequentist analysis. The stop rules will be based on the probability calculations conditional on the data of an OR>1, corresponding to a non-zero effect but also of an OR>1.05 corresponding to a more marked effect, but also on the probabilities of OR<1 ineffectiveness or of a marked deleterious effect OR < 0.95 with respective probability thresholds set at 0.95,0.80,0.8,0.75. Vague priors but adapted to Bayesian studies using ORs will be used. The maximum size of the study is set at N=500 patients (based on the size of a frequentist study with a power of 80% to demonstrate a difference of 10%

This document is the property of the DRCI/APHP. Any reproduction is strictly prohibited.
Version n°4.0 dated 31/05/2019

between the 2 groups (i.e. 25% vs 15%). The simulations carried out show a high probability that the study will be conclusive after the inclusion of less than 300 patients.

Descriptive statistical analysis:
For each group: The descriptive statistical analysis will include for each quantitative parameter: mean, standard deviation, minimum, maximum, median and quartiles, number of missing values. The qualitative parameters will be expressed by the frequency of distribution.

Bayesian statistics:

Main criterion:
The posterior probabilities of the primary endpoint will be calculated using different types of priors (non-informative or vague).
The following posterior probabilities will be calculated:
P (OR>1), corresponding to a non-zero effect of the prone position.
P (OR>1.05) corresponding to an effect that is already clinically interesting.
P (OR<1) corresponding to inefficiency of the prone position.
P (OR < 0.95) corresponding to a deleterious effect of the prone position.

The thresholds considered particularly interesting for the a posteriori probabilities described below are respectively equal to 0.95, 0.80, 0.8, 0.75.

Secondary Criteria
For each parameter, the a posteriori probabilities will be described using different types of priors (non-informative or vague). The 95% credibility intervals will be calculated.

Secondary analyses
An analysis of the following subpopulations is planned a priori:
- Age >70 years
- Patients with NIV or high-flow oxygenation at baseline
- Patients included in intensive care or not

Statistical criteria for stopping the search.
Interim analyses will be done every 50 patients (followed up to the primary endpoint).
The study may be stopped for efficacy after the 2nd interim analysis, if one of the 2 conditions below is met:
P(OR>1) >0.95
P (OR>1.05) >0.8
The study may be stopped after the 2nd interim analysis for safety if P (OR< 0.95) >0.75
The study may be stopped for futility after the 4th interim analysis
P(OR>1) < 0.1

Method of accounting for missing, unused or invalid data
All patients included in the database will be analyzed. The reasons for leaving the study and/or protocol violations will be described for each group. An analysis of patients who have followed the protocol without major violations will be done (per-protocol analysis). It will be considered secondary. For analyses requiring full data in a patient, two analyses will be performed, one on the actual available data, the other using a multiple imputation replacement procedure. The robustness of the

This document is the property of the DRCI/APHP. Any reproduction is strictly prohibited.
Version n°4.0 dated 31/05/2019

conclusions will be verified, and any discrepancies will be analyzed according to the data collection biases that may have been identified.

Manage changes to the initial strategy analysis plan.

In the event of changes to the criteria described in the protocol, a specific statistical analysis plan will be drawn up before any unblinding.

Population Selection

All randomized patients will be analyzed in ITT

A per-protocol analysis on all randomized patients without major violations will be done and considered secondary. A secondary analysis will also be performed according to the duration of the prone position. If the difference between the ITT population and the PP population is < 10%, only the ITT analysis will be performed.

14 QUALITY CONTROL AND ASSURANCE

14.1 General organization

The sponsor must ensure the safety and respect of the individuals who have agreed to participate in the research. It must set up a quality assurance system to monitor the progress of research in the investigator centres as well as possible.

To this end, the promoter will define a strategy for opening up the centres and may, if necessary, set up a data quality control.

These notions will be adapted throughout the confinement period.

14.1.1 Strategy for opening up centres

The strategy for opening the centres will be determined before the start of the research.

14.1.2 Data quality control

In the context of the COVID-19 pandemic, the usual rules for tracking searches cannot be followed. The confinement decreed by the government imposes a degraded operation of the research that is about to begin (APHP promoter), among other things, the impossibility of carrying out monitoring visits on site.

The following procedure aims to define a course of action to be taken in order to adapt the monitoring rules in accordance with rights, the safety of people and the quality of data, always in accordance with the regulations in force. These actions to be taken are defined according to the risk and the level of monitoring of this study, of which the APHP is sponsoring.

This modus operandi will no longer be applied once the confinement period is over.

A Clinical Research Associate (CRA) mandated by the sponsor will ensure that the research is carried out properly by adapting it to the current working conditions (confinement), and to the risk of the study

- Obtaining compliant consents
- Advancing recruitment in research
- Safety of people included in the research
- Update on compliance with the protocol

This document is the property of the DRCI/APHP. Any reproduction is strictly prohibited.
Version n°4.0 dated 31/05/2019

- A strategy for reporting non-conformities (consent and follow-up of recruitment) will be put in place

The investigator and the members of his team agree to make themselves available during the Quality Control visits carried out by telephone at regular intervals by the Clinical Research Associate.

14.2 Case Report Book

The case report form contains only the data necessary for analysis for publication. All the data relating to the subject and necessary for his follow-up during and outside the research will be collected in his medical file.

Paper CRF:
All the information required by the protocol must be recorded in the case report forms and an explanation must be provided for each missing data. The data will have to be collected as they are obtained, and transcribed in these notebooks in a clear and legible way.
Erroneous data found in the case report forms will be crossed out and the new data will be copied, next to the crossed out information, accompanied by the initials, the date and possibly a justification by the investigator or the authorized person who made the correction.

Electronic FIU :
All information required by the protocol should be recorded in the case report forms. Data will need to be collected as they are obtained, and recorded in these notebooks explicitly. Each missing data will have to be coded.
This electronic observation notebook will be set up in each of the centres using an Internet data collection medium. A guidance document for the use of this tool will be provided to investigators.
The investigator is responsible for the accuracy, quality, and relevance of all data entered. In addition, when they are entered, this data is immediately checked for consistency. In this regard, the investigator must validate any change in value in the CRF. These changes are subject to an audit trail. A justification may be included in the commentary.
A paper printout will be requested at the end of the study, authenticated (dated and signed) by the investigator. The original of this document will be archived by the proponent. A copy of the authenticated document should be archived by the investigator.

14.3 Non-conformance management

Any event occurring as a result of non-compliance with the protocol, standard operating procedures, or applicable laws and regulations by an investigator or any other person involved in the conduct of the research must be declared non-compliant to the sponsor.

These non-conformities will be managed in accordance with the proponent's procedures.

14.4 Audit

The investigators undertake to accept the quality assurance audits carried out by the sponsor as well as the inspections carried out by the competent authorities. All data, documents and reports can be audited and regulated without medical confidentiality being invoked.

This document is the property of the DRCI/APHP. Any reproduction is strictly prohibited.
Version n°4.0 dated 31/05/2019

An audit may be carried out at any time by persons mandated by the sponsor and independent of the research managers. Its objective is to ensure the quality of the research, the validity of its results and compliance with the law and regulations in force.

Individuals who direct and monitor the research agree to comply with the sponsor's requirements for an audit
The audit may apply to all stages of research, from the development of the protocol to the publication of the results and the classification of the data used or produced in the research.

14.5 Commitment of responsibilities of the Principal Investigator

In the current conditions of confinement and overload of work in the care services, it will be accepted to have available at the beginning of the study a CV dated more than one year, unsigned and/or undated to be updated by each investigator as soon as this period is lifted. The same goes for training courses related to clinical research.

Each investigator will undertake to comply with legislative and regulatory obligations and to conduct research in accordance with the regulations, in accordance with the terms of the Declaration of Helsinki in force.

The principal investigator of each participating centre will sign a commitment of responsibilities (DRCI type document) which will be given to the sponsor's representative.
Investigators and their collaborators will sign a delegation of duties form specifying the role of each and provide their CV.

15 ETHICAL AND LEGAL ASPECTS

15.1 Procedures for informing and obtaining the consent of persons participating in the research

In accordance with Article L. 1122-1-1 of the Public Health Code, no research involving a human person with a risk and minimal constraint may be carried out on a person without his or her free and informed consent, and expressly after the information provided for in Article L. 1122-1 of the same Code has been delivered.

A reflection period adapted to the COVID-19 pathology will be given to the person between the time he or she is informed and the time he or she signs the consent form, as the positioning must begin as soon as possible.

The free, informed and written consent of the person is obtained by the principal investigator, or by a doctor who represents him/her, before the inclusion (before any research-related acts) of the person in the research, and this as early as possible in the COVID patient care department (list of participating centers in addendum) on presentation of the written information note and oral explanations.
After both parties have signed, the person will keep the briefing note describing the research and the consent and the principal investigator or the physician representing them will keep a copy of the consent signed by the person. The briefing note is available throughout the research in the investigator workbook. It will be updated in the workbook if it changes.

This document is the property of the DRCI/APHP. Any reproduction is strictly prohibited.
Version n°4.0 dated 31/05/2019

A copy will be placed at the end of the study in a sealed tamper-proof envelope containing all the consent forms, which will be archived by the sponsor.

In addition, the investigator will specify in the person's medical record the person's participation in the research, the procedures for obtaining his or her consent and the procedures for providing the information for the purpose of obtaining it. He or she keeps a copy of the form for the collection of consent of the person, dated and signed.

15.2 Prohibition on participation in any other research or exclusion period provided for at the end of the research, if applicable

During the period of participation in the research, the patient may participate in another research protocol involving the human person. The latter, included in this study with minimal risks in COVID services, are asked to participate in several protocols. The principal investigator or his representative will verify that participation in two research projects at the same time is without any interaction with each other while ensuring maximum patient safety, in order to advance the research as quickly as possible.
Patients will also be able to participate in other non-interventional research, if they agree.

15.3 Legal obligations

The Assistance publique hôpitaux de Paris (AP-HP) is the promoter of this research and by delegation the Clinical Research and Innovation Department (DRCI) carries out its missions, in accordance with Article L.1121-1 of the Public Health Code. Assistance Publique - Hôpitaux de Paris reserves the right to interrupt the research at any time for medical or administrative reasons; in this case, a notification will be provided to the investigator

15.4 Request for an opinion from the Committee for the Protection of Persons CPP

The AP-HP, as a sponsor, obtains the favourable opinion of the CPP concerned for intervention research with minimal risks and constraints, prior to its implementation, within the framework of its competences and in accordance with the legislative and regulatory provisions in force.

15.5 Information from the ANSM

The sponsor AP-HP will send the CPP's favourable opinion and the summary of the protocol for information to the ANSM.

15.6 Procedures relating to the Data Protection Regulations

The computer file used for this research is implemented in accordance with French regulations (amended Data Protection Act) and European regulations (General Data Protection Regulation – GDPR).

- Commitment to comply with the "Reference Methodology" MR 001

This research falls within the framework of the "Reference Methodology for the processing of personal data implemented in the context of research in the field of health" (MR-001 as amended). AP-HP, the promoter of the research, has signed a commitment to comply with this "Reference Methodology"

This document is the property of the DRCI/APHP. Any reproduction is strictly prohibited.
Version n°4.0 dated 31/05/2019

15.7 Research Changes

Any substantial changes made to the protocol by the coordinating investigator should be forwarded to the sponsor for approval. After this agreement, the promoter must obtain a favourable opinion from the CPP prior to its implementation

The information note and the consent form may be revised if necessary, in particular in the event of a substantial change in the search or the occurrence of adverse effects.

15.8 Final Research Report

The final report of research involving humans referred to in Article R1123-67 of the CSP is drawn up and signed by the sponsor and the investigator. A summary of the report drawn up in accordance with the reference plan of the competent authority shall be submitted to the competent authority within one year after the end of the research, corresponding to the end of the participation of the last person who participates in the research.

15.9 Archiving

The specific documents of a research involving the human person at risk and minimal constraints will be archived by the investigator and the sponsor for a period of 15 years after the end of the research.

This indexed archiving includes:

- A sealed investigator envelope containing an original copy of all information notes and signed consent forms of all individuals at the research centre;
- A sealed envelope for the sponsor containing a copy of all briefing notes and signed consent forms for all individuals at the site who participated in the research;
- The "research" workbooks for the Investigator and the sponsor include (non-exhaustive list):
    - the successive versions of the protocol (identified by the number and date of version), its annexes
    - the opinions of the CPP
    - correspondence letters,
    - the list or register of inclusion,
    - research-specific annexes
    - the final report of the research.
- Data collection documents.

16 FINANCING AND INSURANCE

16.1 Source of funding

COVID-19 INNOVARC project.

This document is the property of the DRCI/APHP. Any reproduction is strictly prohibited.
Version n°4.0 dated 31/05/2019

16.2 Insurance

The Sponsor takes out insurance for the entire duration of the research guaranteeing its own civil liability as well as that of any doctor involved in the conduct of the research. It also ensures full compensation for the harmful consequences of the research for the person who participates in it and his or her beneficiaries, unless he or she can prove that the damage is not attributable to his or her fault or that of any participant, without it being possible to oppose the act of a third party or the voluntary withdrawal of the person who had initially consented to the research.

The Assistance Publique-Hôpitaux de Paris (AP-HP) has taken out insurance with the company HDI-GLOBAL SE through BIOMEDIC-INSURE, guaranteeing its civil liability as well as that of any participant (doctor or staff involved in carrying out the research), in accordance with Article L.1121-10 of the CSP.

17 PUBLICATION RULES

17.1 Mention of the AP-HP's affiliation for projects promoted by the AP-HP

- If an author has several affiliations, the order in which the institutions are cited (AP-HP, University, INSERM, etc.) does not matter
- However, if the research is funded through an internal call for tenders from the AP-HP, the first affiliation should be "AP-HP"
- Each of these affiliations must be identified by an address separated by a semicolon (;
- The AP-HP institution must appear under the acronym "**AP-HP**" first in the address followed precisely by: **AP-HP**, hospital, department, city, postal code, France

17.2 Mention of the AP-HP promoter (DRCI) in the manuscript's acknowledgments

- "The sponsor was Assistance Publique – Hôpitaux de Paris (Clinical Research and Innovation Department)"

17.3 Mention of the funder in the manuscript's acknowledgments

INNOVARC COVID-19 Project April 2020

The research is registered on clinicaltrials.gov under the number NCT04366856

18 BIBLIOGRAPHY

1. Guérin C, Reignier J, Richard J-C, Beuret P, Gacouin A, Boulain T, Mercier E, Badet M, Mercat A, Baudin O, Clavel M, Chatellier D, Jaber S, Rosselli S, Mancebo J, Sirodot M, Hilbert G, Bengler C, Richecoeur J, Gainnier M, Bayle F, Bourdin G, Leray V, Girard R, Baboi L, Ayzac L: Prone Positioning in Severe Acute Respiratory Distress Syndrome. New England Journal of Medicine 2013; 368:2159–68
2. Wu C, Chen X, Cai Y, Xia J, Zhou X, Xu S, Huang H, Zhang L, Zhou X, Du C, Zhang Y, Song J, Wang S, Chao Y, Yang Z, Xu J, Zhou X, Chen D, Xiong W, Xu L, Zhou F, Jiang J, Bai C, Zheng J, Song Y: Risk Factors Associated With Acute Respiratory Distress Syndrome and Death in Patients With Coronavirus Disease 2019 Pneumonia in Wuhan, China. JAMA Internal Medicine 2020 doi:10.1001/jamainternmed.2020.0994

This document is the property of the DRCI/APHP. Any reproduction is strictly prohibited.
Version n°4.0 dated 31/05/2019

3. Alhazzani W, Møller MH, Arabi YM, Loeb M, Gong MN, Fan E, Oczkowski S, Levy MM, Derde L, Dzierba A, Du B, Aboodi M, Wunsch H, Cecconi M, Koh Y, Chertow DS, Maitland K, Alshamsi F, Belley-Cote E, Greco M, Laundy M, Morgan JS, Kesecioglu J, McGeer A, Mermel L, Mammen MJ, Alexander PE, Arrington A, Centofanti JE, Citerio G, et al.: Surviving Sepsis Campaign: guidelines on the management of critically ill adults with Coronavirus Disease 2019 (COVID-19). Intensive Care Medicine 2020 doi:10.1007/s00134-020-06022-5
4. World Health Organisation: Clinical management of severe acute respiratory infection (SARI) when COVID-19 disease is suspected. 2020
5. Wang D, Hu B, Hu C, Zhu F, Liu X, Zhang J, Wang B, Xiang H, Cheng Z, Xiong Y, Zhao Y, Li Y, Wang X, Peng Z: Clinical Characteristics of 138 Hospitalized Patients With 2019 Novel Coronavirus–Infected Pneumonia in Wuhan, China. JAMA 2020; 323:1061
6. Guan W, Ni Z, Hu Y, Liang W, or C, He J, Liu L, Shan H, Lei C, Hui DSC, Du B, Li L, Zeng G, Yuen K-y, Chen R, Tang C, Wang T, Chen P, Xiang J, Li S, Wang J, Liang Z, Peng Y, Wei L, Liu Y, Hu Y, Peng P, Wang J, Liu J, Chen Z, et al.: Clinical Characteristics of Coronavirus Disease 2019 in China. New England Journal of Medicine 2020 doi:10.1056/NEJMoa2002032
7. Young BE, Ong SWX, Kalimuddin S, Low JG, Tan SY, Loh J, Ng O-T, Marimuthu K, Ang LW, Mak TM, Lau SK, Anderson DE, Chan KS, Tan TY, Ng TY, Cui L, Said Z, Kurupatham L, Chen MI-C, Chan M, Vasoo S, Wang L-F, Tan BH, Lin RTP, Lee VJM, Leo Y-S, Lye DC, for the Singapore 2019 Novel Coronavirus Outbreak Research Team: Epidemiologic Features and Clinical Course of Patients Infected With SARS-CoV-2 in Singapore. JAMA 2020 doi:10.1001/jama.2020.3204

This document is the property of the DRCI/APHP. Any reproduction is strictly prohibited.

19 LIST OF PROTOCOL ADDENDA

19.1 List of Investigators Addendum number 1

Centre	Adresse du centre			Investigateur Principal
001	Hôpital du Kremlin Bicêtre	Service de pneumologie	78, rue du général Leclerc 94275 Le Kremlin Bicêtre	Dr Laurent SAVALE
002	Hôpital du Kremlin Bicêtre	Service de médecine Interne	78, rue du général Leclerc 94275 Le Kremlin Bicêtre	Dr Nicolas NOEL
003	Hôpital du Kremlin Bicêtre	Service de maladies infectieuses	78, rue du général Leclerc 94275 Le Kremlin Bicêtre	Pr Stephane JAUREGUIBERRY
004	Hôpital du Kremlin Bicêtre	Service Réanimation Pédiatrique Accueil Adultes	78, rue du général Leclerc 94275 Le Kremlin Bicêtre	Dr Philippe DURAND
005	Hôpital du Kremlin Bicêtre	Service de réanimation chirurgicale	78, rue du général Leclerc 94275 Le Kremlin Bicêtre	Dr Anatole HARROIS
006	Hôpital Ambroise Paré	Service de pneumologie	9, Avenue Charles de Gaulle 92100 Boulogne-Billancourt	Pr Thierry CHINET
007	Hôpital Ambroise Paré	Service de réanimation	9, Avenue Charles de Gaulle 92100 Boulogne-Billancourt	Pr Antoine VIEILLARD-BARON
008	Hôpital Paul Brousse	Service d'hépathologie (accueil adultes COVID)	12, Avenue Paul Vaillant-Couturier	Dr Audrey COILLY
009	Hôpital Paul Brousse	Service de réanimation	12, Avenue Paul Vaillant-Couturier	Dr Philippe ICHAI
010	Hôpital George Pompidou	Service de Pneumologie	20, rue Leblanc, 75015 Paris	Dr Olivier SANCHEZ
011	Hôpital Lariboisière	Service Anesthésie-Réanimation	2, rue Ambroise Paré 75475 Paris Cedex 10	Dr Etienne GAYAT
012	Hôpital Lariboisière	Médecine interne	2, rue Ambroise Paré 75475 Paris Cedex 10	Pr Damien SENE
013	Hôpital Lariboisière	Endocrinologie	2, rue Ambroise Paré 75475 Paris Cedex 10	Pr Jean-François GAUTIER
014	Hôpital Antoine Béclère	Service de Gériatrie	157, rue de la Porte Trivaux 92140 Clamart	Dr Mathieu MION
015	Hôpital Antoine Béclère	Service de médecine interne-Infectiologie	157, rue de la Porte Trivaux 92140 Clamart	Dr Dorothée VIGNES
016	Hôpital Antoine Béclère	Service de Réanimation	157, rue de la Porte Trivaux 92140 Clamart	Dr Charles DAMOISEL
017	Hôpital des Armées	Service Anesthésie-Réanimation	101 avenue Henri Barbusse BP 406 – 92141 Clamart Cedex	Dr Stéphane DE RUDNICKI
018	Hôpital des Armées	Service de Médecine Interne	101 avenue Henri Barbusse BP 406 – 92141 Clamart Cedex	Dr Marc ALETTI
019	Hôpital Avicenne	Service de Réanimation	125, rue de Stalingrad 93000 Bobigny	Pr Stéphane GAUDRY
020	Hôpital Avicenne	Service de Pneumologie	125, rue de Stalingrad 93000 Bobigny	Pr Hilario NUNES
021	Hôpital Avicenne	Service de maladies Infectieuses	125, rue de Stalingrad 93000 Bobigny	Pr Olivier BOUCHAUD

This document is the property of the DRCI/APHP. Any reproduction is strictly prohibited.
Version n°4.0 dated 31/05/2019

19.2 Patient collection form Addendum number 2

Patient PIN Label

Dear Madam, Sir, you have agreed to participate in the PROVID research and we thank you for that.

The doctor in charge of this study has explained the procedure to follow

You will stand on your stomach with your head turned to the side and a cushion placed under your chest at your convenience for at least 6 hours daily with consecutive two-hour periods if you can.
Every day you will have to note on this support the time spent in the position, a pen is at your disposal
The collection begins from the moment you have agreed to participate in the study by signing a consent

Data collection on ➔ I__I__I__I/I__I__I/2020 from the signing of the consent at 8 a.m. (7:59 a.m.) the next day

Session n°1 I__I__Ih I__I__Imn **Session n°4** I__I__Ih I__I__Imn **Session n°7** I__I__Ih I__I__Imn
Session n°2 I__I__Ih I__I__Imn **Session n°5** I__I__Ih I__I__Imn **Session n°8** I__I__Ih I__I__Imn
Session n°3 I__I__Ih I__I__Imn **Session n°6** I__I__Ih I__I__Imn **Session n°9** I__I__Ih I__I__Imn
TOTAL: I__I__Ih I__I__Imn

Day 2 = I__I__I__I/I__I__I/2020 Data collection from 8:00 am to 8:00 am (7:59 am) to be collected throughout the day

Session n°1 I__I__Ih I__I__Imn **Session n°4** I__I__Ih I__I__Imn **Session n°7** I__I__Ih I__I__Imn
Session n°2 I__I__Ih I__I__Imn **Session n°5** I__I__Ih I__I__Imn **Session n°8** I__I__Ih I__I__Imn
Session n°3 I__I__Ih I__I__Imn **Session n°6** I__I__Ih I__I__Imn **Session n°9** I__I__Ih I__I__Imn
TOTAL: I__I__Ih I__I__Imn

Day 3 = I__I__I__I/I__I__I/2020 Data collection from 8:00 am to 8:00 am (7:59 am) to be collected throughout the day

Session n°1 I__I__Ih I__I__Imn **Session n°4** I__I__Ih I__I__Imn **Session n°7** I__I__Ih I__I__Imn
Session n°2 I__I__Ih I__I__Imn **Session n°5** I__I__Ih I__I__Imn **Session n°8** I__I__Ih I__I__Imn
Session n°3 I__I__Ih I__I__Imn **Session n°6** I__I__Ih I__I__Imn **Session n°9** I__I__Ih I__I__Imn
TOTAL: I__I__Ih I__I__Imn

Day 4 = I__I__I__I/I__I__I/2020 Data collection from 8:00 a.m. to 8:00 a.m. (7:59 a.m.) to be collected throughout the day

Session n°1 I__I__Ih I__I__Imn **Session n°4** I__I__Ih I__I__Imn **Session n°7** I__I__Ih I__I__Imn
Session n°2 I__I__Ih I__I__Imn **Session n°5** I__I__Ih I__I__Imn **Session n°8** I__I__Ih I__I__Imn
Session n°3 I__I__Ih I__I__Imn **Session n°6** I__I__Ih I__I__Imn **Session n°9** I__I__Ih I__I__Imn
TOTAL: I__I__Ih I__I__Imn

Day 5 = I__I__I__I/I__I__I/2020 Data collection from 8:00 am to 8:00 am (7:59 am) to be collected throughout the day

Session n°1 I__I__IhI__I__Imn **Session n°4** I__I__IhI__I__Imn **Session n°7** I__I__IhI__I__Imn

Session n°2 I__I__IhI__I__Imn **Session n°5** I__I__IhI__I__Imn **Session n°8** I__I__IhI__I__Imn

Session n°3 I__I__IhI__I__Imn **Session n°6** I__I__IhI__I__Imn **Session n°9** I__I__IhI__I__Imn

TOTAL: I__I__IhI__I__Imn

Day 6 = I__I__I__I/I__I__I/2020 Data collection from 8:00 am to 8:00 am (7:59 am) to be collected throughout the day

Session n°1 I__I__IhI__I__Imn **Session n°4** I__I__IhI__I__Imn **Session n°7** I__I__IhI__I__Imn

Session n°2 I__I__IhI__I__Imn **Session n°5** I__I__IhI__I__Imn **Session n°8** I__I__IhI__I__Imn

Session n°3 I__I__IhI__I__Imn **Session n°6** I__I__IhI__I__Imn **Session n°9** I__I__IhI__I__Imn

TOTAL: I__I__IhI__I__Imn

Day 7 = I__I__I__I/I__I__I/2020 Data collection from 8:00 am to 8:00 am (7:59 am) to be collected throughout the day

Session n°1 I__I__IhI__I__Imn **Session n°4** I__I__IhI__I__Imn **Session n°7** I__I__IhI__I__Imn

Session n°2 I__I__IhI__I__Imn **Session n°5** I__I__IhI__I__Imn **Session n°8** I__I__IhI__I__Imn

Session n°3 I__I__IhI__I__Imn **Session n°6** I__I__IhI__I__Imn **Session n°9** I__I__IhI__I__Imn

TOTAL: I__I__IhI__I__Imn

Day 8 = I__I__I__I/I__I__I/2020 Data collection from 8:00 am to 8:00 am (7:59 am) to be collected throughout the day

Session n°1 I__I__IhI__I__Imn **Session n°4** I__I__IhI__I__Imn **Session n°7** I__I__IhI__I__Imn

Session n°2 I__I__IhI__I__Imn **Session n°5** I__I__IhI__I__Imn **Session n°8** I__I__IhI__I__Imn

Session n°3 I__I__IhI__I__Imn **Session n°6** I__I__IhI__I__Imn **Session n°9** I__I__IhI__I__Imn

TOTAL: I__I__IhI__I__Imn

Day 9 = I__I__I__I/I__I__I/2020 Data collection from 8:00 am to 8:00 am (7:59 am) to be collected

throughout the day

Session n°1 I__I__IhI__I__Imn **Session n°4** I__I__IhI__I__Imn **Session n°7** I__I__IhI__I__Imn

Session n°2 I__I__IhI__I__Imn **Session n°5** I__I__IhI__I__Imn **Session n°8** I__I__IhI__I__Imn

Session n°3 I__I__IhI__I__Imn **Session n°6** I__I__IhI__I__Imn **Session n°9** I__I__IhI__I__Imn

TOTAL: I__I__IhI__I__Imn

PROVID study: Effects of prone positioning in patients on spontaneous ventilation with COVID 19 hypoxemic lung disease
Patient identification : Centre I__I__I__I / Inclusion order number I__I__I__I__I Patient initials I__I/I__I

Day 10 = I__I__I__I/I__I__I/2020 Data collection from 8:00 a.m. to 8:00 a.m. (7:59 a.m.) **to be collected throughout the day**

Session n°1 I__I__Ih I__I__Imn **Session n°4** I__I__Ih I__I__Imn **Session n°7** I__I__Ih I__I__Imn

Session n°2 I__I__Ih I__I__Imn **Session n°5** I__I__Ih I__I__Imn **Session n°8** I__I__Ih I__I__Imn

Session n°3 I__I__Ih I__I__Imn **Session n°6** I__I__Ih I__I__Imn **Session n°9** I__I__Ih I__I__Imn

TOTAL: I__I__Ih I__I__Imn

Day 11 = I__I__I__I/I__I__I/2020 Data collection from 8:00 am to 8:00 am (7:59 am) **to be collected throughout the day**

Session n°1 I__I__Ih I__I__Imn **Session n°4** I__I__Ih I__I__Imn **Session n°7** I__I__Ih I__I__Imn

Session n°2 I__I__Ih I__I__Imn **Session n°5** I__I__Ih I__I__Imn **Session n°8** I__I__Ih I__I__Imn

Session n°3 I__I__Ih I__I__Imn **Session n°6** I__I__Ih I__I__Imn **Session n°9** I__I__Ih I__I__Imn

TOTAL: I__I__Ih I__I__Imn

Day 12 = I__I__I__I/I__I__I/2020 Data collection from 8:00 a.m. to 8:00 a.m. (7:59 a.m.) **to be collected throughout the day**

Session n°1 I__I__Ih I__I__Imn **Session n°4** I__I__Ih I__I__Imn **Session n°7** I__I__Ih I__I__Imn

Session n°2 I__I__Ih I__I__Imn **Session n°5** I__I__Ih I__I__Imn **Session n°8** I__I__Ih I__I__Imn

Session n°3 I__I__Ih I__I__Imn **Session n°6** I__I__Ih I__I__Imn **Session n°9** I__I__Ih I__I__Imn

TOTAL: I__I__Ih I__I__Imn

Day 13 = I__I__I__I/I__I__I/2020 Data collection from 8:00 am to 8:00 am (7:59 am) **to be collected throughout the day**

Session n°1 I__I__Ih I__I__Imn **Session n°4** I__I__Ih I__I__Imn **Session n°7** I__I__Ih I__I__Imn

Session n°2 I__I__Ih I__I__Imn **Session n°5** I__I__Ih I__I__Imn **Session n°8** I__I__Ih I__I__Imn

Session n°3 I__I__Ih I__I__Imn **Session n°6** I__I__Ih I__I__Imn **Session n°9** I__I__Ih I__I__Imn

TOTAL: I__I__Ih I__I__Imn

PROVID study: Effects of prone positioning in patients on spontaneous ventilation with COVID 19 hypoxemic lung disease
Patient identification : Centre I__I__I__I / Inclusion order number I__I__I__I__I Patient initials I__I/I__I

Day 14 = I__I__I__I/I__I__I/2020 Data collection from 8:00 a.m. to 8:00 a.m. (7:59 a.m.) to be collected throughout the day

Session n°1 I__I__IhI__I__Imn **Session n°4** I__I__IhI__I__Imn **Session n°7** I__I__IhI__I__Imn

Session n°2 I__I__IhI__I__Imn **Session n°5** I__I__IhI__I__Imn **Session n°8** I__I__IhI__I__Imn

Session n°3 I__I__IhI__I__Imn **Session n°6** I__I__IhI__I__Imn **Session n°9** I__I__IhI__I__Imn

TOTAL: I__I__IhI__I__Imn

Day 15 = I__I__I__I/I__I__I/2020 Data collection from 8:00 am to 8:00 am (7:59 am) to be collected throughout the day

Session n°1 I__I__Ih I__I__Imn **Session n°4** I__I__Ih I__I__Imn **Session n°7** I__I__Ih I__I__Imn

Session n°2 I__I__Ih I__I__Imn **Session n°5** I__I__Ih I__I__Imn **Session n°8** I__I__Ih I__I__Imn

Session n°3 I__I__Ih I__I__Imn **Session n°6** I__I__Ih I__I__Imn **Session n°9** I__I__Ih I__I__Imn

TOTAL: I__I__Ih I__I__Imn

Day 16 = I__I__I__I/I__I__I/2020 Data collection from 8:00 am to 8:00 am (7:59 am) to be collected throughout the day

Session n°1 I__I__Ih I__I__Imn **Session n°4** I__I__Ih I__I__Imn **Session n°7** I__I__Ih I__I__Imn

Session n°2 I__I__Ih I__I__Imn **Session n°5** I__I__Ih I__I__Imn **Session n°8** I__I__Ih I__I__Imn

Session n°3 I__I__Ih I__I__Imn **Session n°6** I__I__Ih I__I__Imn **Session n°9** I__I__Ih I__I__Imn

TOTAL: I__I__Ih I__I__Imn

Day 17 = I__I__I__I/I__I__I/2020 Data collection from 8:00 am to 8:00 am (7:59 am) to be collected throughout the day

Session n°1 I__I__Ih I__I__Imn **Session n°4** I__I__Ih I__I__Imn **Session n°7** I__I__Ih I__I__Imn

Session n°2 I__I__Ih I__I__Imn **Session n°5** I__I__Ih I__I__Imn **Session n°8** I__I__Ih I__I__Imn

Session n°3 I__I__Ih I__I__Imn **Session n°6** I__I__Ih I__I__Imn **Session n°9** I__I__Ih I__I__Imn

TOTAL: I__I__Ih I__I__Imn

Day 18 = I__I__I__I/I__I__I/2020 Data collection from 8:00 am to 8:00 am (7:59 am) to be collected throughout the day

Session n°1 I__I__Ih I__I__Imn **Session n°4** I__I__Ih I__I__Imn **Session n°7** I__I__Ih I__I__Imn

Session n°2 I__I__Ih I__I__Imn **Session n°5** I__I__Ih I__I__Imn **Session n°8** I__I__Ih I__I__Imn

Session n°3 I__I__Ih I__I__Imn **Session n°6** I__I__Ih I__I__Imn **Session n°9** I__I__Ih I__I__Imn

TOTAL: I__I__Ih I__I__Imn

Day 19 = I__I__I__I/I__I__I/2020 Data collection from 8:00 a.m. to 8:00 a.m. (7:59 a.m.) to be collected throughout the day

Session n°1 I__I__IhI__I__Imn **Session n°4** I__I__IhI__I__Imn **Session n°7** I__I__IhI__I__Imn

Session n°2 I__I__IhI__I__Imn **Session n°5** I__I__IhI__I__Imn **Session n°8** I__I__IhI__I__Imn

Session n°3 I__I__IhI__I__Imn **Session n°6** I__I__IhI__I__Imn **Session n°9** I__I__IhI__I__Imn

TOTAL: I__I__IhI__I__Imn

Day 20 = I__I__I__I/I__I__I/2020 Data collection from 8:00 a.m. to 8:00 a.m. (7:59 a.m.) to be collected throughout the day

Session n°1 I__I__Ihl__I__Imn **Session n°4** I__I__Ihl__I__Imn **Session n°7** I__I__Ihl__I__Imn

Session n°2 I__I__Ihl__I__Imn **Session n°5** I__I__Ihl__I__Imn **Session n°8** I__I__Ihl__I__Imn

Session n°3 I__I__Ihl__I__Imn **Session n°6** I__I__Ihl__I__Imn **Session n°9** I__I__Ihl__I__Imn

TOTAL: I__I__Ihl__I__Imn

Day 21 = I__I__I__I/I__I__I/2020 Data collection from 8:00 am to 8:00 am (7:59 am) to be collected throughout the day

Session n°1 I__I__Ihl__I__Imn **Session n°4** I__I__Ihl__I__Imn **Session n°7** I__I__Ihl__I__Imn

Session n°2 I__I__Ihl__I__Imn **Session n°5** I__I__Ihl__I__Imn **Session n°8** I__I__Ihl__I__Imn

Session n°3 I__I__Ihl__I__Imn **Session n°6** I__I__Ihl__I__Imn **Session n°9** I__I__Ihl__I__Imn

TOTAL: I__I__Ihl__I__Imn

Day 22 = I__I__I__I/I__I__I/2020 Data collection from 8:00 a.m. to 8:00 a.m. (7:59 a.m.) to be collected throughout the day

Session n°1 I__I__Ihl__I__Imn **Session n°4** I__I__Ihl__I__Imn **Session n°7** I__I__Ihl__I__Imn

Session n°2 I__I__Ihl__I__Imn **Session n°5** I__I__Ihl__I__Imn **Session n°8** I__I__Ihl__I__Imn

Session n°3 I__I__Ihl__I__Imn **Session n°6** I__I__Ihl__I__Imn **Session n°9** I__I__Ihl__I__Imn

TOTAL: I__I__Ihl__I__Imn

Day 23 = I__I__I__I/I__I__I/2020 Data collection from 8:00 a.m. to 8:00 a.m. (7:59 a.m.) to be collected throughout the day

Session n°1 I__I__Ihl__I__Imn **Session n°4** I__I__Ihl__I__Imn **Session n°7** I__I__Ihl__I__Imn

Session n°2 I__I__Ihl__I__Imn **Session n°5** I__I__Ihl__I__Imn **Session n°8** I__I__Ihl__I__Imn

Session n°3 I__I__Ihl__I__Imn **Session n°6** I__I__Ihl__I__Imn **Session n°9** I__I__Ihl__I__Imn

TOTAL: I__I__Ihl__I__Imn

Day 24 = I__I__I__I/I__I__I/2020 Data collection from 8:00 a.m. to 8:00 a.m. (7:59 a.m.) to be collected throughout the day

Session n°1 I__I__Ihl__I__Imn **Session n°4** I__I__Ihl__I__Imn **Session n°7** I__I__Ihl__I__Imn

Session n°2 I__I__Ihl__I__Imn **Session n°5** I__I__Ihl__I__Imn **Session n°8** I__I__Ihl__I__Imn

Session n°3 I__I__Ihl__I__Imn **Session n°6** I__I__Ihl__I__Imn **Session n°9** I__I__Ihl__I__Imn

TOTAL: I__I__Ihl__I__Imn

PROVID study: Effects of prone positioning in patients on spontaneous ventilation with COVID 19 hypoxemic lung disease
Patient identification : Centre I__I__I__I / Inclusion order number I__I__I__I__I Patient initials I__I/I__I

Day 25 = I__I__I__I/I__I__I/2020 Data collection from 8:00 am to 8:00 am (7:59 am) to be collected throughout the day

Session n°1 I__I__Ih I__I__Imn **Session n°4** I__I__Ih I__I__Imn **Session n°7** I__I__Ih I__I__Imn
Session n°2 I__I__Ih I__I__Imn **Session n°5** I__I__Ih I__I__Imn **Session n°8** I__I__Ih I__I__Imn
Session n°3 I__I__Ih I__I__Imn **Session n°6** I__I__Ih I__I__Imn **Session n°9** I__I__Ih I__I__Imn

TOTAL: I__I__Ih I__I__Imn

Day 26 = I__I__I__I/I__I__I/2020 Data collection from 8:00 am to 8:00 am (7:59 am) to be collected throughout the day

Session n°1 I__I__Ih I__I__Imn **Session n°4** I__I__Ih I__I__Imn **Session n°7** I__I__Ih I__I__Imn
Session n°2 I__I__Ih I__I__Imn **Session n°5** I__I__Ih I__I__Imn **Session n°8** I__I__Ih I__I__Imn
Session n°3 I__I__Ih I__I__Imn **Session n°6** I__I__Ih I__I__Imn **Session n°9** I__I__Ih I__I__Imn

TOTAL: I__I__Ih I__I__Imn

Day 27 = I__I__I__I/I__I__I/2020 Data collection from 8:00 a.m. to 8:00 a.m. (7:59 a.m.) to be collected throughout the day

Session n°1 I__I__Ih I__I__Imn **Session n°4** I__I__Ih I__I__Imn **Session n°7** I__I__Ih I__I__Imn
Session n°2 I__I__Ih I__I__Imn **Session n°5** I__I__Ih I__I__Imn **Session n°8** I__I__Ih I__I__Imn
Session n°3 I__I__Ih I__I__Imn **Session n°6** I__I__Ih I__I__Imn **Session n°9** I__I__Ih I__I__Imn

TOTAL: I__I__Ih I__I__Imn

Day 28 = I__I__I__I/I__I__I/2020 Data collection from 8:00 am to 8:00 am (7:59 am) to be collected throughout the day

Session n°1 I__I__Ih I__I__Imn **Session n°4** I__I__Ih I__I__Imn **Session n°7** I__I__Ih I__I__Imn
Session n°2 I__I__Ih I__I__Imn **Session n°5** I__I__Ih I__I__Imn **Session n°8**

I__I__IhI__I__Imn

Session n°3 I__I__IhI__I__Imn **Session n°6** I__I__IhI__I__Imn **Session n°9** I__I__IhI__I__Imn

TOTAL: I__I__IhI__I__Imn

End of the collection.

Thank you for your participation
