# Supplementary material for: Awake Prone Positioning in Patients With COVID-19 Respiratory Failure: A Randomized Clinical Trial
Source: JAMA Netw Open. 2025 Dec 10;8(12):e2548201. doi: 10.1001/jamanetworkopen.2025.48201 (PMC12696593; doi:10.1001/jamanetworkopen.2025.48201)

Supplemental Online Content

Harrois A, Jouffroy R, Ayed S, et al. Awake prone positioning in patients with COVID-19 respiratory failure: a randomized clinical trial. *JAMA Netw Open*. 2025;8(12):e2548201. doi:10.1001/jamanetworkopen.2025.48201

eTable 1. Use of Immunomodulators and Antiviral Drugs in the Intention-to-Treat Population
eTable 2. Probability of Treatment Effects Estimated by Bayesian Analysis According to Different Prior Beliefs About 'Intubation and/or Death' Benefit From Prone Positioning in Patients in the Intention-to-Treat Population
eTable 3. Probability of Treatment Effects Estimated by Bayesian Analysis According to Different Prior Beliefs About 'Days Alive and Free From Mechanical Ventilation' Benefit From Prone Positioning in the Intention-to-Treat Population
eTable 4. Probability of Treatment Effects Estimated by Bayesian Analysis According to Different Prior Beliefs About 'Days Alive Outside ICU' Benefit From Prone Positioning in the Intention-to-Treat Population
eTable 5. Probability of Treatment Effects Estimated by Bayesian Analysis According to Different Prior Beliefs About 'Days Alive Outside the Hospital' Benefit From Prone Positioning in the Intention-to-Treat Population
eTable 6. Probability of Treatment Effects Estimated by Bayesian Analysis According to Different Prior Beliefs About 'ICU Admission' Benefit From Prone Positioning in the Intention-to-Treat Population
eTable 7. Probability of Treatment Effects Estimated by Bayesian Analysis According to Different Prior Beliefs About 'Intubation and/or Death' Benefit From Prone Positioning in the Per Protocol Population
eTable 8. Probability of Treatment Effects Estimated by Bayesian Analysis According to Different Prior Beliefs About 'Days Alive and Free From Mechanical Ventilation' Benefit From Prone Positioning in the Per Protocol Population
eTable 9. Probability of Treatment Effects Estimated by Bayesian Analysis According to Different Prior Beliefs About 'Days Alive Outside ICU' Benefit From Prone Positioning in the Per Protocol Population
eTable 10. Probability of Treatment Effects Estimated by Bayesian Analysis According to Different Prior Beliefs About 'Days Alive Outside the Hospital' Benefit From Prone Positioning in the Per Protocol Population

eTable 11. Probability of Treatment Effects Estimated by Bayesian Analysis According to Different Prior Beliefs About 'ICU Admission' Benefit From Prone Positioning in the Per Protocol Population

eTable 12. Probability of Treatment Effects Estimated by Bayesian Analysis According to Different Prior Beliefs About 'Intubation and/or Death' Benefit From Prone Positioning in the Subgroup of Patients Admitted in ICU at the Time of Enrolment

eTable 13. Probability of Treatment Effects Estimated by Bayesian Analysis According to Different Prior Beliefs About 'Intubation and/or Death' Benefit From Prone Positioning in the Subgroup of Patients Admitted in the Ward (Not in ICU) at the Time of Enrolment

eTable 14. Probability of Treatment Effects Estimated by Bayesian Analysis According to a Non-informative Prior About 'Intubation and/or Death' Benefit From Prone Positioning in Patients According to their BMI

eFigure. Kaplan Meier Survival Curve for A/ Tracheal Intubation and/or Death B/ Tracheal Intubation, C/ Death Within 28 Days of Enrollment in the Intention-to-Treat Population

This supplemental material has been provided by the authors to give readers additional information about their work.

eTable 1: Use of immunomodulators and antiviral drugs in the intention-to treat-population

**Drug**	**Standard** group N=221	**Awake Prone positioning** group N=223
**Corticosteroids, n(%)**	218 (99)	223 (100)
**Tocilizumab, n(%)**	69 (31)	77 (34)
**Remdesivir, n(%)**	3 (1)	1 (0)

eTable 2: Probability of treatment effects estimated by Bayesian analysis according to different prior beliefs about 'Intubation and/or death' benefit from prone positioning in patients in the intention-to-treat population

Prior belief	Mean odds ratio OR (95% CI)	Posterior Probability OR < or > given threshold, %				
		OR<1	OR<0·95	OR<0·9	OR>1	OR>1·05
**Non-Informative**	0·74(0·48-1·09)	93·83	90·18	84·84	6·17	3·81
**Optimistic Moderate**	0·72(0·48-1·04)	96·18	93·54	89·28	3·82	2·19
**Optimistic Strong**	0·69(0·47-0·97)	98·24	96·65	93·82	1·76	0·87
**Skeptical Moderate**	0·80(0·56-1·12)	90·79	85·07	76·95	9·21	5·47
**Skeptical Strong**	0·86(0·64-1·14)	86·06	76·76	63·96	13·94	7·80

eTable 3: Probability of treatment effects estimated by Bayesian analysis according to different prior beliefs about 'Days alive and free from mechanical ventilation' benefit from prone positioning in the intention-to treat population

**Prior belief**	**Mean difference MD (95% CI)**	**Sd**	**Posterior Probability MD < or > given threshold, %**				
			**Mean < -2**	**Mean < -1**	**Mean > 0**	**Mean > 1**	**Mean > 2**
**Non-Informative**	0·33(-1·37, 2·03)	0·87	0·35	6·19	64·90	22·00	2·72
**Optimistic Moderate**	0·62(-0·94, 2·17)	0·79	0·05	2·09	78·24	31·30	4·03
**Optimistic Strong**	0·90(-0·49, 2·29)	0·71	0·00	0·36	89·83	44·20	5·95
**Skeptical Moderate**	0·05(-0·60, 0·69)	0·33	0·00	0·07	55·98	0·19	0·00
**Skeptical Strong**	0·02(-0·37, 0·41)	0·20	0·00	0·00	53·57	0·00	0·00

eTable 4: Probability of treatment effects estimated by Bayesian analysis according to different prior beliefs about 'Days alive outside ICU' benefit from prone positioning in the intention-to treat population

Prior belief	Mean difference MD (95% CI)	Sd	Posterior Probability MD < or > given threshold, %				
			Mean < -2	Mean < -1	Mean > 0	Mean > 1	Mean > 2
**Non-Informative**	1·28(-0·78, 3·34)	1·05	0·09	1·51	88·77	60·39	24·53
**Optimistic Moderate**	1·45(-0·36, 3·27)	0·92	0·01	0·41	94·20	68·77	27·62
**Optimistic Strong**	1·59(0·03, 3·15)	0·80	0·00	0·06	97·72	77·16	30·54
**Skeptical Moderate**	0·13(-0·53, 0·79)	0·34	0·00	0·04	65·26	0·49	0·00
**Skeptical Strong**	0·05(-0·35, 0·44)	0·20	0·00	0·00	59·17	0·00	0·00

eTable 5: Probability of treatment effects estimated by Bayesian analysis according to different prior beliefs about 'Days alive outside the hospital' benefit from prone positioning in the intention-to treat population

Prior belief	Mean difference MD (95% CI)	Sd	Posterior Probability MD < or > given threshold, %				
			Mean < -2	Mean < -1	Mean > 0	Mean > 1	Mean > 2
**Non-Informative**	1·55(-0·22, 3·32)	0·90	0	0·24	95·69	72·90	30·86
**Optimistic Moderate**	1·64(0·04, 3·25)	0·82	0	0·07	97·78	78·37	33·01
**Optimistic Strong**	1·72(0·30, 3·14)	0·72	0	0·01	99·13	83·86	34·84
**Skeptical Moderate**	0·21(-0·44, 0·85)	0·33	0	0·01	73·55	0·82	0·00
**Skeptical Strong**	0·08(-0·31, 0·47)	0·20	0	0·00	64·94	0·00	0·00

eTable 6: Probability of treatment effects estimated by Bayesian analysis according to different prior beliefs about 'ICU admission' benefit from prone positioning in the intention-to treat-population

Prior belief	Mean odds ratio OR (95% CI)	Posterior Probability OR < or > given threshold, %				
		OR<1	OR<0.95	OR<0.9	OR>1	OR>1.05
**Non-Informative**	0.99(0.44-1.91)	58.92	53.47	47.65	41.08	36.07
**Optimistic Moderate**	0.84(0.42-1.51)	75.83	70.60	64.69	24.17	19.72
**Optimistic Strong**	0.73(0.40-1.22)	89.60	85.91	81.18	10.40	7.58
**Skeptical Moderate**	0.99(0.58-1.59)	56.20	48.24	39.89	43.80	36.50
**Skeptical Strong**	1(0.69-1.39)	54.17	42.92	31.51	45.83	35.23

eTable 7: Probability of treatment effects estimated by Bayesian analysis according to different prior beliefs about 'Intubation and/or death' benefit from prone positioning in the per protocol population

Prior belief	Mean odds ratio OR (95% CI)	Posterior Probability OR < or > given threshold, %				
		OR<1	OR<0·95	OR<0·9	OR>1	OR>1·05
**Non-Informative**	0·58(0·35-0·89)	99·26	98·70	97·70	0·74	0·40
**Optimistic Moderate**	0·57(0·35-0·86)	99·60	99·22	98·51	0·40	0·21
**Optimistic Strong**	0·56(0·36-0·82)	99·83	99·64	99·23	0·17	0·07
**Skeptical Moderate**	0·68(0·45-0·99)	97·84	96·11	93·18	2·16	1·17
**Skeptical Strong**	0·79(0·58-1·06)	94·39	89·58	81·90	5·61	2·83

eTable 8: Probability of treatment effects estimated by Bayesian analysis according to different prior beliefs about 'Days alive and free from mechanical ventilation' benefit from prone positioning in the per protocol population

Prior belief	Mean difference MD (95% CI)	Sd	Posterior Probability MD < or > given threshold, %				
			Mean < -2	Mean < -1	Mean > 0	Mean > 1	Mean > 2
**Non-Informative**	0·77(-1·07, 2·61)	0·94	0·16	2·99	79·36	40·13	9·41
**Optimistic Moderate**	1·01(-0·65, 2·67)	0·85	0·02	0·87	88·31	50·39	12·15
**Optimistic Strong**	1·23(-0·22, 2·69)	0·74	0·00	0·14	95·11	62·28	15·07
**Skeptical Moderate**	0·1(-0·55, 0·75)	0·33	0·00	0·04	61·47	0·32	0·00
**Skeptical Strong**	0·04(-0·36, 0·43)	0·20	0·00	0·00	56·89	0·00	0·00

eTable 9: Probability of treatment effects estimated by Bayesian analysis according to different prior beliefs about 'Days alive outside ICU' benefit from prone positioning in the per protocol population

Prior belief	Mean difference MD (95% CI)	Sd	Posterior Probability MD < or > given threshold, %				
			Mean < -2	Mean < -1	Mean > 0	Mean > 1	Mean > 2
**Non-Informative**	1·40(-0·85, 3·64)	1·15	0·18	1·85	88·89	63·55	29·91
**Optimistic Moderate**	1·57(-0·37, 3·50)	0·99	0·02	0·48	94·43	71·80	33·11
**Optimistic Strong**	1·69(0·05, 3·34)	0·84	0·00	0·07	97·83	79·55	35·74
**Skeptical Moderate**	0·12(-0·54, 0·79)	0·34	0·00	0·05	64·04	0·49	0·00
**Skeptical Strong**	0·04(-0·35, 0·44)	0·20	0·00	0·00	58·57	0·00	0·00

eTable 10: Probability of treatment effects estimated by Bayesian analysis according to different prior beliefs about 'Days alive outside the hospital' benefit from prone positioning in the per protocol population

Prior belief	Mean difference MD (95% CI)	Sd	Posterior Probability MD < or > given threshold, %				
			Mean < -2	Mean < -1	Mean > 0	Mean > 1	Mean > 2
**Non-Informative**	1·40(-0·57, 3·36)	1·00	0·03	0·85	91·85	65·40	27·15
**Optimistic Moderate**	1·54(-0·20, 3·28)	0·89	0·00	0·22	95·80	72·63	30·11
**Optimistic Strong**	1·65(0·13, 3·17)	0·78	0·00	0·03	98·36	79·85	32·56
**Skeptical Moderate**	0·16(-0·50, 0·81)	0·34	0·00	0·03	68·00	0·60	0·00
**Skeptical Strong**	0·06(-0·34, 0·45)	0·20	0·00	0·00	60·88	0·00	0·00

eTable 11: Probability of treatment effects estimated by Bayesian analysis according to different prior beliefs about 'ICU admission' benefit from prone positioning in the per protocol population

**Prior belief**	**Mean odds ratio OR (95% CI)**	**Posterior Probability OR < or > given threshold, %**				
		**OR<1**	**OR<0·95**	**OR<0·9**	**OR>1**	**OR>1·05**
**Non-Informative**	0·99(0·40-2·06)	59·38	54·50	49·29	40·62	36·13
**Optimistic Moderate**	0·82(0·38-1·53)	77·38	72·74	67·38	22·62	18·68
**Optimistic Strong**	0·71(0·37-1·21)	90·70	87·48	83·34	9·30	6·83
**Skeptical Moderate**	1(0·56-1·63)	56·04	48·48	40·59	43·96	36·96
**Skeptical Strong**	1(0·68-1·41)	54·11	43·11	31·92	45·89	35·63

eTable 12: Probability of treatment effects estimated by Bayesian analysis according to different prior beliefs about 'Intubation and/or death' benefit from prone positioning in the subgroup of patients admitted in ICU at the time of enrolment

**Prior belief**	**Mean odds ratio OR (95% CI)**	**Posterior Probability OR < or > given threshold, %**				
		**OR<1**	**OR<0·95**	**OR<0·9**	**OR>1**	**OR>1·05**
**Non-Informative**	0·77(0·47-1·19)	88·85	84·04	77·80	11·15	7·70
**Optimistic Moderate**	0·74(0·46-1·11)	93·25	89·72	84·60	6·75	4·33
**Optimistic Strong**	0·70(0·45-1·02)	96·88	94·67	91·16	3·12	1·79
**Skeptical Moderate**	0·84(0·56-1·2)	84·55	77·39	68·34	15·45	10·23
**Skeptical Strong**	0·89(0·65-1·2)	78·84	67·99	54·68	21·16	13·18

eTable 13: Probability of treatment effects estimated by Bayesian analysis according to different prior beliefs about 'Intubation and/or death' benefit from prone positioning in the subgroup of patients admitted in the ward (not in ICU) at the time of enrolment

Prior belief	Mean odds ratio OR (95% CI)	Posterior Probability OR < or > given threshold, %				
		OR<1	OR<0·95	OR<0·9	OR>1	OR>1·05
**Non-Informative**	0·66(0·23-1·48)	87·06	84·61	81·67	12·94	10·86
**Optimistic Moderate**	0·60(0·26-1·18)	93·51	91·63	89·25	6·49	5·01
**Optimistic Strong**	0·57(0·29-1·00)	97·57	96·47	94·89	2·43	1·68
**Skeptical Moderate**	0·86(0·47-1·43)	75·19	69·07	62·00	24·81	19·70
**Skeptical Strong**	0·94(0·64-1·33)	67·42	56·96	45·51	32·58	23·85

eTable 14. probability of treatment effects estimated by Bayesian analysis according to a non-informative prior about 'Intubation and/or death' benefit from prone positioning in patients according to their BMI

ITT Analysis	subgroup: BMI < 30						subgroup: BMI ≥ 30					
**Outcome**	**Standard group** N=137	**Awake Prone positioning group** N=127	**Mean OR (95% CI)**	**Posterior Probability of OR < 1 (%)**	**Mean estimated difference (95% CI)**	**Posterior probability of a difference (%)**	**Standard group** N= 81	**Awake Prone positioning group** N= 92	**Mean OR (95% CI)**	**Posterior Probability of OR < 1 (%)**	**Mean estimated difference (95% CI)**	**Posterior probability of a difference (%)**
Primary outcome												
Intubation and/or death, n(%)	46 (34)	32 (25)	0.69 (0.39 to 1.13)	93.35	NA	NA	27 (33)	27 (29)	0.88 (0.43 to 1.59)	71.37	NA	NA
Intubation, n(%)	39 (28)	29 (23)	0.77 (0.42 to 1.3)	85.34	NA	NA	26 (32)	25 (27)	0.83 (0.41 to 1.52)	76.10	NA	NA
Death, n(%)	22 (16)	13 (10)	0.63 (0.28 to 1.23)	92.02	NA	NA	11 (14)	12 (13)	1.06 (0.4 to 2.33)	53.91	NA	NA
Secondary outcomes												
Days alive and free from mechanical ventilation (28d), days	23.44; 28 (26 to 28)	23.73; 28 (28 to 28)	NA	NA	0.29 (-1.82 to 2.40)	60.69	22.39; 28 (20 to 28)	22.6; 28 (24 to 28)	NA	NA	0.21 (-2.65 to 3.08)	55.73
Days alive outside ICU (28d), days	15.96; 21 (0 to 25)	17.43; 22 (6 to 26)	NA	NA	1.45 (-1.20 to 4.11)	85.81	16.09; 21 (0 to 25)	16.97; 22 (1 to 25)	NA	NA	0.86 (-2.45 to 4.17)	69.37
Days alive outside the hospital (28d), days	10.04; 11 (0 to 20)	12.43; 16 (0 to 21)	NA	NA	2.35 (0.06 to 4.65)	97.75	12.14; 16 (0 to 20)	12.18; 16 (0 to 20)	NA	NA	0.05 (-2.79 to 2.88)	51.25
ICU admission (when not in ICU at enrolment), n(%)	10 (24)*	5 (15)*	0.67 (0.16 to 1.8)	83.36	NA	NA	6 (29)*	9 (33)*	1.58 (0.36 to 4.64)	35.47	NA	NA

Data are n(%), mean; median (IQR). CI: credible interval, OR: odds ratio.

For patients with BMI <30: *42 patients in the standard group and 33 patients in the awake prone positioning group were not admitted into ICU at the time of enrolment into the study.

For patients with BMI ≥30: *21 patients in the standard group and 27 patients in the awake prone positioning group were not admitted into ICU at the time of enrolment into the study.

eFigure 1 : Kaplan Meier survival curve for A/ tracheal intubation and/or death B/ tracheal intubation, C/ death within 28 days of enrollment in the intention-to treat-population.

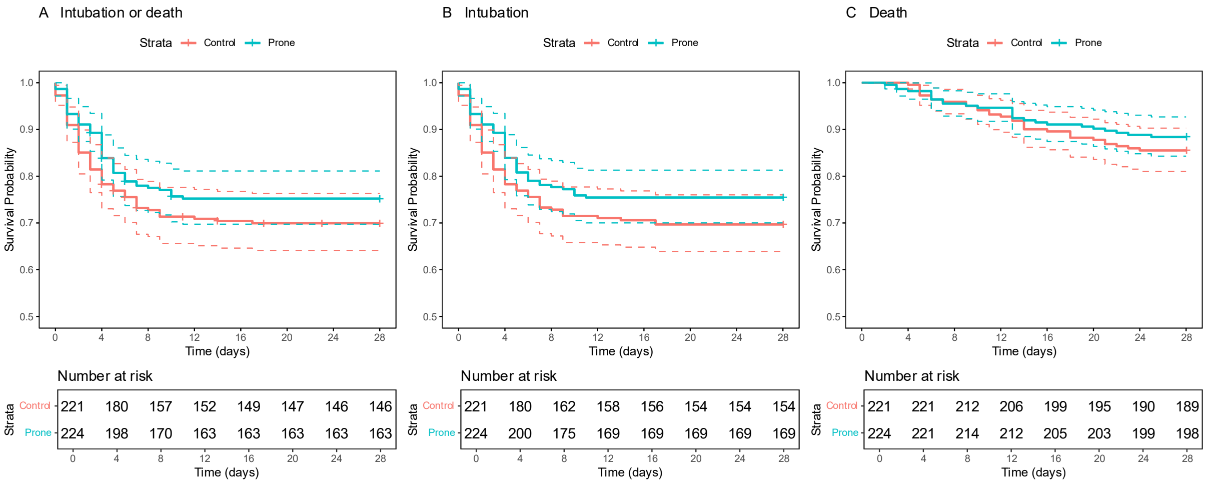

Supplement: Supplement 2. — eTable 1. Use of Immunomodulators and Antiviral Drugs in the Intention-to-Treat Population eTable 2. Probability of Treatment Effects Estimated by Bayesian Analysis According to Different Prior Beliefs About 'Intubation and/or Death' Benefit From Prone Positioning in Patients in the Intention-to-Treat Population eTable 3. Probability of Treatment Effects Estimated by Bayesian Analysis According to Different Prior Beliefs About 'Days Alive and Free From Mechanical Ventilation' Benefit From Prone Positioning in the Intention-to-Treat Population eTable 4. Probability of Treatment Effects Estimated by Bayesian Analysis According to Different Prior Beliefs About 'Days Alive Outside ICU' Benefit From Prone Positioning in the Intention-to-Treat Population eTable 5. Probability of Treatment Effects Estimated by Bayesian Analysis According to Different Prior Beliefs About 'Days Alive Outside the Hospital' Benefit From Prone Positioning in the Intention-to-Treat Population eTable 6. Probability of Treatment Effects Estimated by Bayesian Analysis According to Different Prior Beliefs About 'ICU Admission' Benefit From Prone Positioning in the Intention-to-Treat Population eTable 7. Probability of Treatment Effects Estimated by Bayesian Analysis According to Different Prior Beliefs About 'Intubation and/or Death' Benefit From Prone Positioning in the Per Protocol Population eTable 8. Probability of Treatment Effects Estimated by Bayesian Analysis According to Different Prior Beliefs About 'Days Alive and Free From Mechanical Ventilation' Benefit From Prone Positioning in the Per Protocol Population eTable 9. Probability of Treatment Effects Estimated by Bayesian Analysis According to Different Prior Beliefs About 'Days Alive Outside ICU' Benefit From Prone Positioning in the Per Protocol Population eTable 10. Probability of Treatment Effects Estimated by Bayesian Analysis According to Different Prior Beliefs About 'Days Alive Outside the Hospital' Benefit From Prone Positioning [file jamanetwopen-e2548201-s002.pdf]
