# Supplementary material for: Awake Prone Positioning in Patients With COVID-19 Respiratory Failure: A Randomized Clinical Trial
Source: JAMA Netw Open. 2025 Dec 10;8(12):e2548201. doi: 10.1001/jamanetworkopen.2025.48201 (PMC12696593; doi:10.1001/jamanetworkopen.2025.48201)
Supplement: Supplement 3. — PROVID Study Group [file jamanetwopen-e2548201-s003.pdf]

Supplemental Online Content: Nonauthor Collaborators
*First name, last name, and suffix (if applicable) are required and will appear in PubMed.

*Group Name(s): PROVID Study Group							
***First Name and Middle Initial(s)**	***Last Name**	***Suffix (eg, Jr, III)**	Academic Degrees	Institution	Location (city, state/province, country)	Role or Contribution, eg, chair, principal investigator	Group (if more than 1 Group listed in the byline) and/or Subgroup (eg, Steering Committee)
Edouard	Jullien		MD	Medical ICU, Ambroise Paré Hospital, Assistance Publique-Hôpitaux de Paris	Boulogne-Billancourt, Ile de France, France	Investigator	
Quentin	Fosse		MD	Medical ICU, Bicêtre Hospital, Assistance Publique-Hôpitaux de Paris	Kremlin Bicêtre, Ile de France, France	Investigator	
Christopher	Lai		MD	Medical ICU, Bicêtre Hospital, Assistance Publique-Hôpitaux de Paris	Kremlin Bicêtre, Ile de France, France	Investigator	
Arthur	Pavot		MD	Medical ICU, Bicêtre Hospital, Assistance Publique-Hôpitaux de Paris	Kremlin Bicêtre, Ile de France, France	Investigator	
Xavier	Pavot		MD	Medical ICU, Bicêtre Hospital, Assistance Publique-Hôpitaux de Paris	Kremlin Bicêtre, Ile de France, France	Investigator	
Thai	Pham		MD	Medical ICU, Bicêtre Hospital, Assistance Publique-Hôpitaux de Paris	Kremlin Bicêtre, Ile de France, France	Investigator	
Candice	Fontaine		MD	Medical ICU, Groupe Hospitalier Pa	Paris , Ile de France, France	Investigator	
Christian	Laplace		MD	Surgical ICU, Bicêtre Hospital, Assistance Publique-Hôpitaux de Paris	Kremlin Bicêtre, Ile de France, France	Investigator	
Gaelle	Cheisson		MD	Surgical ICU, Bicêtre Hospital, Assistance Publique-Hôpitaux de Paris	Kremlin Bicêtre, Ile de France, France	Investigator	
Benjamin	Bergis		MD	Surgical ICU, Bicêtre Hospital, Assistance Publique-Hôpitaux de Paris	Kremlin Bicêtre, Ile de France, France	Investigator	
Jonas	Pochard		MD	Surgical ICU, Bicêtre Hospital, Assistance Publique-Hôpitaux de Paris	Kremlin Bicêtre, Ile de France, France	Investigator	

Supplemental Online Content: Nonauthor Collaborators
*First name, last name, and suffix (if applicable) are required and will appear in PubMed.

***First Name and Middle Initial(s)**	***Last Name**	***Suffix (eg, Jr, III)**	Academic Degrees	Institution	Location (city, state/province, country)	Role or Contribution, eg, chair, principal investigator	Group (if more than 1 Group listed in the byline) and/or Subgroup (eg, Steering Committee)
Aurore	Rodrigues		MD	Surgical ICU, Bicêtre Hospital, Assistance Publique-Hôpitaux de Paris	Kremlin Bicêtre, Ile de France, France	Investigator	
Pierre-Etienne	Leblanc		MD	Surgical ICU, Bicêtre Hospital, Assistance Publique-Hôpitaux de Paris	Kremlin Bicêtre, Ile de France, France	Investigator	
Samy	Figueiredo		MD	Surgical ICU, Bicêtre Hospital, Assistance Publique-Hôpitaux de Paris	Kremlin Bicêtre, Ile de France, France	Investigator	
Lucille	Wildenberg		MD	Surgical ICU, Bicêtre Hospital, Assistance Publique-Hôpitaux de Paris	Kremlin Bicêtre, Ile de France, France	Investigator	
David	Montani		MD	Pulmonology and respiratory ICU, Bicêtre Hospital, Assistance Publique-Hôpitaux de Paris	Kremlin Bicêtre, Ile de France, France	Investigator	
Marc	Humbert		MD, PhD	Pulmonology and respiratory ICU, Bicêtre Hospital, Assistance Publique-Hôpitaux de Paris	Kremlin Bicêtre, Ile de France, France	Investigator	
Sophioe	Bulifon		MD	Pulmonology and respiratory ICU, Bicêtre Hospital, Assistance Publique-Hôpitaux de Paris	Kremlin Bicêtre, Ile de France, France	Investigator	
Xavier	Jais		MD, PhD	Pulmonology and respiratory ICU, Bicêtre Hospital, Assistance Publique-Hôpitaux de Paris	Kremlin Bicêtre, Ile de France, France	Investigator	
Hélène	Masson		MD	Internal Medicine, Saint Germain en Laye intercommunal Hospital	Saint Germain en Laye, Ile de France, Fran	Investigator	
Catherine	Veyssier-Belot		MD	Internal Medicine, Saint Germain en Laye intercommunal Hospital	Saint Germain en Laye, Ile de France, Fran	Investigator	

Supplemental Online Content: Nonauthor Collaborators
*First name, last name, and suffix (if applicable) are required and will appear in PubMed.

***First Name and Middle Initial(s)**	***Last Name**	***Suffix (eg, Jr, III)**	Academic Degrees	Institution	Location (city, state/province, country)	Role or Contribution, eg, chair, principal investigator	Group (if more than 1 Group listed in the byline) and/or Subgroup (eg, Steering Committee)
Alice	Berezne		MD	Infectious disease - internal medicine department, Annecy Gennevois Hospital	Annecy, Haute Savoie, France	Investigator	
Cécile	Janssen		MD	Infectious disease - internal medicine department, Annecy Gennevois Hospital	Annecy, Haute Savoie, France	Investigator	
Mathilde	Neuville		MD	Medical and surgical ICU, Foch Hospital	Suresnes, Haut de Seine, France	Investigator	
Alexis	Paternot		MD	Medical and surgical ICU, Foch Hospital	Suresnes, Haut de Seine, France	Investigator	
Julia	Egbeola		MD	Medical and surgical ICU, Foch Hospital	Suresnes, Haut de Seine, France	Investigator	
Johana	Cohen		MD	Medical and surgical ICU, Foch Hospital	Suresnes, Haut de Seine, France	Investigator	
Richard	Galliot		MD	Medical and surgical ICU, Foch Hospital	Suresnes, Haut de Seine, France	Investigator	
Selim	Aboulethar		MD	Medical and surgical ICU, Foch Hospital	Suresnes, Haut de Seine, France	Investigator	
Camille	Vassord Dang		MD	Medical and surgical ICU, Foch Hospital	Suresnes, Haut de Seine, France	Investigator	
Jérôme	Devaquet		MD	Medical and surgical ICU, Foch Hospital	Suresnes, Haut de Seine, France	Investigator	
Benjamin	Zuber		MD	Medical and surgical ICU, Foch Hospital	Suresnes, Haut de Seine, France	Investigator	
Stéphane	Gaudry		MD, PhD	Medical ICU, Avicenne Hospital, assistance Pubique Hôpitaux de Paris	Bobigny, Seine Saint Denis, France	Investigator	
Delphine	Ceraudo		MD	Medical ICU, Avicenne Hospital, assistance Pubique Hôpitaux de Paris	Bobigny, Seine Saint Denis, France	Investigator	
Khalil	Chaibi		MD	Medical ICU, Avicenne Hospital, assistance Pubique Hôpitaux de Paris	Bobigny, Seine Saint Denis, France	Investigator	

Supplemental Online Content: Nonauthor Collaborators
*First name, last name, and suffix (if applicable) are required and will appear in PubMed.

***First Name and Middle Initial(s)**	***Last Name**	***Suffix (eg, Jr, III)**	Academic Degrees	Institution	Location (city, state/province, country)	Role or Contribution, eg, chair, principal investigator	Group (if more than 1 Group listed in the byline) and/or Subgroup (eg, Steering Committee)
Nicolas	Bonnet		MD	Medical ICU, Avicenne Hospital, assistance Pubique Hôpitaux de Paris	Bobigny, Seine Saint Denis, France	Investigator	
Guillaume	Van Der Meersch		MD	Medical ICU, Avicenne Hospital, assistance Pubique Hôpitaux de Paris	Bobigny, Seine Saint Denis, France	Investigator	
Johanna	Oziel		MD	Medical ICU, Avicenne Hospital, assistance Pubique Hôpitaux de Paris	Bobigny, Seine Saint Denis, France	Investigator	
Jesus	Vidal-Mayo		MD	Department of Emergency Medicine, Instituto Nacional de Ciencias Medicas y Nutricion Salvador Zubiran	Mexico city, Mexico	Investigator	
Rosario	Hernandez-Ortega		MD	Department of Emergency Medicine, Instituto Nacional de Ciencias Medicas y Nutricion Salvador Zubiran	Mexico city, Mexico	Investigator	
Jose	Jimenez-Ceja		MD	Department of Emergency Medicine, Instituto Nacional de Ciencias Medicas y Nutricion Salvador Zubiran	Mexico city, Mexico	Investigator	
Marie	Lebouc		MD	Anesthesiology and Critical Care Department, Annecy Hospital	Annecy, Haute Savoie, France	Investigator	
Stéphane	Jaureguiberry		MD, PhD	Department of Infectious Diseases, Hôpital de Bicêtre, assistance Pubique Hôpitaux de Paris	Kremlin Bicêtre, Ile de France, France	Investigator	
Sergio	Salmeron		MD	Respiratory medicine, Groupe Hospitalier Paris Saint-Joseph	Paris , Ile de France, France	Investigator	
Hilario	Nunes		MD, PhD	Respiratory medicine, Avicenne Hospital, assistance Pubique Hôpitaux de Paris	Bobigny, Seine Saint Denis, France	Investigator	

Supplemental Online Content: Nonauthor Collaborators
*First name, last name, and suffix (if applicable) are required and will appear in PubMed.

***First Name and Middle Initial(s)**	***Last Name**	***Suffix (eg, Jr, III)**	Academic Degrees	Institution	Location (city, state/province, country)	Role or Contribution, eg, chair, principal investigator	Group (if more than 1 Group listed in the byline) and/or Subgroup (eg, Steering Committee)
Olivier	Bouchaud		MD, PhD	Infectious Disease Department, Avicenne Hospital, assistance Pubique Hôpitaux de Paris	Bobigny, Seine Saint Denis, France	Investigator	
Frédéric	Mechaï		MD	Infectious Disease Department, Avicenne Hospital, assistance Pubique Hôpitaux de Paris	Bobigny, Seine Saint Denis, France	Investigator	
Jeanne	Goupil De Bouille		MD	Infectious Disease Department, Avicenne Hospital, assistance Pubique Hôpitaux de Paris	Bobigny, Seine Saint Denis, France	Investigator	
Gersende	Favé		MD	Anaesthesiology and Critical Care Medicine Department, Hôpital Européen Georges Pompidou, assistance Pubique Hôpitaux de Paris	Paris, Ile de France, France	Investigator	
Bernard	Cholley		MD, PhD	Anaesthesiology and Critical Care Medicine Department, Hôpital Européen Georges Pompidou, assistance Pubique Hôpitaux de Paris	Paris, Ile de France, France	Investigator	
