# Supplementary material for: Awake Prone Positioning in Patients With COVID-19 Respiratory Failure: A Randomized Clinical Trial
Source: JAMA Netw Open. 2025 Dec 10;8(12):e2548201. doi: 10.1001/jamanetworkopen.2025.48201 (PMC12696593; doi:10.1001/jamanetworkopen.2025.48201)
Supplement: Supplement 4. — Data Sharing Statement [file jamanetwopen-e2548201-s004.pdf]

Data Sharing Statement

Harrois. Awake Prone Positioning in Patients With COVID-19 Respiratory Failure. *JAMA Netw Open*. Published December 10, 2025. doi:10.1001/jamanetworkopen.2025.48201

Data
Additional Information: ClinicalTrials.gov, NCT04366856
Data available: No

Additional Information
Explanation for why data not available: Available under condition
